# A novel inducible mutagenesis screen enables to isolate and clone both embryonic and adult zebrafish mutants

**DOI:** 10.1038/s41598-017-10968-w

**Published:** 2017-09-04

**Authors:** Zhipeng Ma, Peipei Zhu, Meijun Pang, Liwei Guo, Nannan Chang, Jiyuan Zheng, Xiaojun Zhu, Ce Gao, Honghui Huang, Zongbin Cui, Jing-Wei Xiong, Jinrong Peng, Jun Chen

**Affiliations:** 1 Key laboratory for Molecular Animal Nutrition, Ministry of Education, Innovation Center for Signaling Network, College of Life Sciences, Zhejiang University, 866 Yu Hang Tang Road, Hangzhou, 310058 China; 2 College of Animal Sciences, Zhejiang University, 866 Yu Hang Tang Road, Hangzhou, 310058 China; 3 Institute of Molecular Medicine, Beijing Key Laboratory of Cardiometabolic Molecular Medicine, and State Key Laboratory of Natural and Biomimetic Drugs, Peking University, Beijing, 100871 China; 4 Key Laboratory of Aquatic Biodiversity and Conservation, Institute of Hydrobiology, Chinese Academy of Sciences, 8 Dong Hu Nan Road, Wuhan, Hubei 430072 P. R. China; 5 Key Laboratory of Freshwater Fish Reproduction and Development, Ministry of Education, State Key Laboratory Breeding Base of Eco-Environments and Bio-Resources of the Three Gorges Reservoir Region, School of Life Sciences, Southwest University, 2 Tiansheng Road, Beibei, Chongqing 400715 China

## Abstract

Conventional genetic screens for recessive mutants are inadequate for studying biological processes in the adult vertebrate due to embryonic lethality. Here, we report that a novel inducible mutagenesis system enables to study gene function in both embryonic and adult zebrafish. This system yields genetic mutants with conditional ectopic over- or under-expression of genes in F_1_ heterozygotes by utilizing inducible Tet-On transcriptional activation of sense or anti-sense transcripts from entrapped genes by Tol2 transposase-meditated transgenesis. Pilot screens identified 37 phenotypic mutants displaying embryonic defects (34 lines), adult fin regeneration defects (7 lines), or defects at both stages (4 lines). Combination of various techniques (such as: generating a new mutant allele, injecting gene specific morpholino or mRNA etc) confirms that Dox-induced embryonic abnormalities in 10 mutants are due to dysfunction of entrapped genes; and that Dox-induced under-expression of 6 genes causes abnormal adult fin regeneration. Together, this work presents a powerful mutagenesis system for genetic analysis from zebrafish embryos to adults in particular and other model organisms in general.

## Introduction

Forward genetic screening in model organisms is one of the major strategies for analyzing complex biological processes. In conventional genetic screens, chemicals^1, 2^, ionizing radiation^3^, or insertions of tagged-DNA^4^ are applied to create random mutations in the zebrafish genome. This often results in full or partial loss-of-function mutations but may also occasionally create gain-of-function mutations. Numerous studies have shown that ectopic expression can also be informative towards the understanding of gene function^5, 6^. This concept has been applied in the development of the gain-of-function screening in *Drosophila melanogaster* ^7, 8^ and zebrafish^9^.

Zebrafish (*Danio rerio*) represents an ideal model system for organ regeneration studies, owing to its capability to regenerate most of their organs/tissues^10^. Although conventional genetic approaches have proven to be extremely powerful in identification of early zebrafish developmental mutants, these approaches are, in general, not suitable for studying adult organ regeneration. For example, in the cases where the gene of interest is essential for early development and so its mutation causes embryonic lethality, that would prevent us from studying the gene function in the adult organism (such as its role in organ regeneration)^10^. Therefore, an inducible system that allows the mutant to grow to adulthood normally and the gene of interest can be conditionally altered (down- or up-regulation) is much required. One approach is screening for temperature-sensitive (TS) mutants^11, 12^. However, the low efficiency of obtaining TS mutants has thus far limited the scope of this technique^13^.

It has been found in zebrafish, mice and *Arabidopsis* that, for some genes, abnormal phenotypes are only observed in gene knockdown mutants, but not in genetic knockout mutants^14–17^. One mechanism for this phenomenon is that compensation to buffer against genetic mutations is only induced by deleterious mutations but not by gene knockdown^18^. These findings suggest that in some cases, screening for gene knockdown mutants has its advantages over for gene knockout mutants.

Here, we report the development of an inducible system for dominant mutant screening in both embryonic and adult zebrafish by combining both the Tet-On transcriptional activation system and highly efficient, Tol2-based transgenesis. The principle is: in presence of doxycycline (Dox), if the Tol2-mediated insertion of the Tet-On promoter is oriented in the same transcriptional direction at the 5′ end of a gene, the entrapped gene will be transcribed to achieve ectopic over-expression; if the insertion of the Tet-On promoter is in the opposite direction in the intragenic region of a gene, it will generate antisense RNA that may down-regulate the expression of the entrapped gene through interrupting transcription or splicing or stability of the target mRNA. In absence of Dox, the entrapped gene will continue to function normally. The advantage of this Tet-On system is that the interruption of entrapped gene expression can be easily achieved in heterozygous zebrafish via Dox treatment at any desired life stages. To our knowledge, this represents, at the first time, a genetic screen for mutants in F_1_ heterozygotes, as well as for enabling to study both embryonic and adult phenotypes in zebrafish in particular and other vertebrates in general.

## Results

### Construction of an inducible system for dominant mutant screen

**Figure 1:**
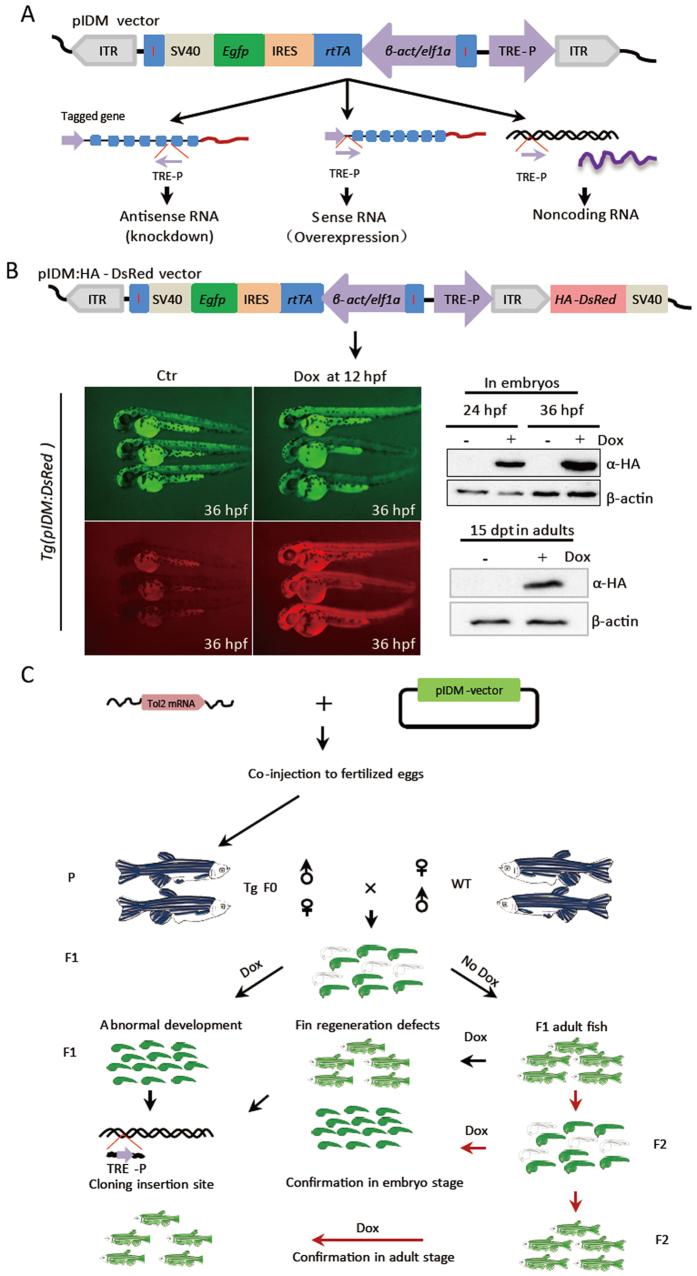
Schematics of the design and strategy of the inducible mutagenesis system for mutant screening. (**A**) Diagram showing the pIDM vector. Upper panel: ITR, inverted terminal repeats of Tol2 transposon; TRE-P, the third generation of tetracycline response element and CMV minimal promoter; I, the chicken β-globin insulator; *β-act*, beta-actin promoter;/elf1a, or elongation factor 1a promoter; rtTA, reverse tetracycline transcriptional activator; IRES, internal ribosome entry site; EGFP, enhanced green fluorescence gene; SV40, the SV40 transcriptional terminator. The purple arrow indicates the orientation of the promoter. Lower panel: upon Dox-treatment, TRE-P transcribes the flanked genomic DNA either antisense RNA (left panel), or sense RNA (middle panel), or non-coding RNA (ncRNA) (right panel), depending upon the inserted position. (**B**) Transgenic line with pIDM carrying a *HA-DsRed* gene and SV40 terminator downstream of the right ITR. The photos were taken at 36 hpf. Tg+/−, heterozygous transgenic fish; Ctr, untreated control sibling. Total protein was extracted at either 24 or 36 hpf. In the transgenic adult fish treated with Dox, total protein was extracted at 15 dpt. An HA monoclonal antibody was used to detect HA-DsRed. β-actin was used as the protein loading control. (**C**) Schematics of the screen strategy. F_0_ transgenic founder fish were crossed with wild type zebrafish (WT) to generate F_1_. F_1_ transgenic embryos in each line were divided into two groups. One group was treated with Dox at 12 hours post fertilization (hpf) to screen mutants with obvious abnormal developmental phenotypes until 5 days post fertilization (dpf). Another group was permitted to grow into adulthood in normal conditions. The 3 month-old F_1_ transgenic fish were treated with Dox 14 days before caudal fin resection. The insertion sites were determined with linker-mediated PCR (LM-PCR) from pooled F_1_ embryos. F_1_ transgenic fish were crossed with WT zebrafish to generate F_2_. All mutants were further confirmed in a heterozygous F_2_ progeny Transgenic fish are shown in green. Red arrows: Confirmation in F_2_ generation.

Tet-On transcriptional activation system has been successfully applied in zebrafish to generate transgenic zebrafish with the conditional expression of interest genes^19, 20^. To facilitate mutant screens and maintenance, we placed the rtTA expression system and the response cis-element (Tet-On promoter) in the same construct to generate a pIDM (plasmid for inducible dominant mutagenesis) vector. The plasmid was comprised of a third generation of tetracycline response element (Tet-On 3 G), a CMV minimal promoter, and a pair of chicken β-globin insulators^21^, as well as an expression cassette including the β-actin (β-act) or elongation factor 1a (elf1α) promoter, the rtTA gene, an internal ribosome entry site (IRES), the EGFP gene, and the SV40 terminator (Fig. 1A, right to left). All of these components were flanked with inverted terminal repeats of Tol2 transposon. Either β-act or elf1α promoter was used to ubiquitously express rtTA (for activating the Tet-on promoter) and EGFP (for transgenic selection).

To analyze whether the right border sequence of Tol2 transposon has any effect on the transcriptional activity of the Tet-On promoter, we placed the HA-tagged DsRed (HA-DsRed) reporter gene plus the SV40 terminator immediately downstream of the right border (Fig. 1B) and generated a transgenic line Tg(pIDM:HA-DsRed). While EGFP was constitutively expressed, DsRed was only induced upon Dox-treatment at either embryonic (whole-embryos and crosections) or adult stage in Tg(pIDM:HA-DsRed) transgenic zebrafish (Figs 1B and S1A,B). This demonstrated that the inducible system worked efficiently in a Dox-dependent manner, having no or little leaky, Dox-independent expression of DsRed.

To decrease position effects on the expression of transgene, we placed an insulator on each side of the expression cassette (Figs 1A and S2). The intensity of EGFP signal was much stronger in most of transgenic lines with the insulators than that in the lines without the insulators (the first generation pIDM) (Fig. S2B). The frequency of F_0_ fish with visible EGFP F_1_ progenies increased to 43% (300/700) as compared to that generated with a plasmid lacking the insulators (25.4% 84/331) (Fig. S2A,B). PCR with a pair of rtTA specific primers showed that the rtTA fragment was also amplified from some F_1_ embryos that lack EGFP signal from the pIDM without insulators (Fig. S2D). The results suggested that these F1 embryos without visible EGFP still contained transgenes. Thus, application of insulators in pIDM indeed decreased position effects on the expression of transgene, rather than increased transgenic frequencies. Interestingly, the leakage of the Tet-On promoter was reduced by including the insulators (Fig. S2E).

To validate the feasibility of this system in a genome-wide scale, we carried out a pilot screen. A total of 300 independent heritable EGFP-positive transgenic lines were generated from 700 F_0_ fish (Fig. S2B). Southern blot analysis on F_1_ embryos of 11 independent transgenic lines showed that the insertion sites in the genome were from 1 to 7 in these founder lines (Fig. S3).

The strategy for the screening of Dox-dependent mutants is outlined in Fig. 1C. In total, 24 lines were found to display Dox-dependent abnormal embryonic development (Table 1; Figs S4–9; S13–15). These 24 lines, and other 95 lines displaying normal development, were then used to screen for caudal fin regeneration defects. In total, 7 fin regeneration mutant lines were identified. The information of identified mutants such as line name, number of insertion, position in target gene, relative level of target gene expression and phenotypes, is summarized in Table 1. Taken together the above design, our pilot-screen data suggest that this pIDM system can be applied for genome-wide mutagenesis screens for both embryonic and adult mutants in zebrafish.

**Table 1:** Information of the pIDM inducible mutants.

Vector-line	Insertion number; Location(linkage group, site, direction)	Relative level of mRNA^a^	Phenotype
pIDM-A11	1;*faf1*(8, 6^th^ intron, reverse)	~55%	FDD^c^; cartilage defects; DFR^d^
pIDM-E10	1;*grb14*(9, 2^nd^ intron of isoform X1, 1st intron of isoform X3, reverse)	isoform X1~30%, isoform X3~13%	Curve body, small head, DFR
pIDM-A1	1;*wu:fb77a09*(17, 9^th^ intron of isoform X1, reverse)	X1-X7~45%	Short stature; DFR
pIDM-A28	2;[*galnt2*(13, 1^st^ intron of *galnt2*, reverse)]; [N.D]	*galnt2*~41%	Short stature; DFR
pIDM-A3	3;[*sema5ba*(9, 15^th^ intron, reverse)];[*plekha5*(4, 12^th^ intron, reverse)];[*LOC101*8*8*2*061*(23,4^th^ intron, reverse)]	*LOC101*8*8206*1~38%	DFR
pIDM-A18	2;[*ttyh3b*(1, 3^rd^ intron, reverse)];[si:dkey-26i13.8(1,1st exon and ~200 bp upstream ATG, forward)]	*ttyh3b~2*.*5 F; si:dkey-26i*13.*8~59%*	DFR
pIDM-E7	3;[*fnip1*(21, 16^th^ intron, reverse)];[*cyr61*(3, 1st intron, reverse)];[N.D]	*cyr61~40%*	DFR
pIDM-E3	1;*pcyt1αa*(2, ~1.0 kb upstream of 1st exon, forward)	~28%	Small eyes with less pigmentation, brain heamorrhage
pIDM-E17	1;*ap2b1*(5, 19^th^ intron, reverse)	~44%	Incomplete epiboly, yolk rupture
pIDM-A200	1;*sgcd*(21, 2^nd^ intron of *sgcd*, 2^nd^ intron of isoformX1, reverse)	~38%	Curve body and pericardial edema, failed heart construction
pIDM-A17	1;*LOC556929*(23, ~1.7 kb upstream of 1st exon, forward)	~3.3 F^b^	Abnormal development with curved body
pIDM-E8	1;*plcd1a*(24, 1st exon and 75 bp upstream of ATG of isoformX1, forward)	~12 F, isoformX1~11 F	No eyes, small brain and curved body
pIDM-A33	3;[*gpr128*(22, 64 bp upstream of 1st exon, forward)]; [*picalmb*(15, 12^th^ intron, reverse)]; [14.165 kb at 5′ site: *Loc10*3*911*4*889*(8, forward)]	*gpr128* ~ 3.34 F; *picalmb* ~ 39.5%	Epidermal blisters
pIDM-E22	2;[*sidkey-256k1*3.2(23, 5′UTR region and ~4.4 kb upstream of ATG, forward)]; [*ms4a17a*.0.*8*(4, ~4.5 kb upstream of 1st exon, forward)]	*sidkey-256k13*.*2*~ 4.15 F; *ms4a17a*.0.*8*~ 6.16 F	Less pigmentation
pIDM-A20	4;[*f2rl1*.*1*(21, ~3.6 kb upstream of 1st exon, forward)]; [*hes6*(2,~2.2 kb downstream of last exon, reverse)]; [*myt1l*(23, 2^nd^ intron, forward)];[*dner*(18, 1st intron, reverse)]	*f2rl1*.*1*~3.69 F; *hes6*,NS;*myt1l*X*1/X2* ~2.19 F; *myt1l*X*3* ~52%; *LOC103909182*(NC) ~ 2.0 F	Early embryonic lethal
pIDM-E54	2;[*syt10*(4, 3^th^ intron, forward)];[N.D]	*syt10*, NS	Shorter and thicker extension
pIDM-E19	2;[*grik4*(15,6^th^ exon, reverse)];[N.D]	*grik4-001*~76%; *grik4-002*(NC),NS; *grik4-003*(NC)~64%; *LOC103908836*(NC) ~73%	Small head, curved body and unabsorbed yolk
pIDM-E1	3;[*LOC103910266*(3, 1st intron, reverse)];[113 bp at 3′ side: *pim-3*(25,reverse)];[*si:ch211-15e22*.*3*(1, 7^th^, intron, forward)]	*syt17* ~84%; *LOC103910266*(NC) ~41%; *si:ch211-15e22*.*3*, NS	Pericardial edema and deformed head
pIDM-E14	2;[*abi1a*(24, 1st intron, reverse)];[*TANC1-like*(9, 1st intron, forward)]	*abi1a* ~39%; *acbd5a* ~15%;*TANC1-like* ~37%; *LOC103911696*(NC) ~34%	Short stature and no pericardium
pIDM-E46	3;[*evi5a*(2, ~3.5 kb upstream of 1st exon, forward)];[*LOC101886424*(4, ~2.6 kb upstream of 1st exon, forward)]; [*LOC324205* (1, 1st intron, reverse)]	*evi5a*~4.38F;*LOC101886424*(NC)~1.97F; *LOC324205*,NS; *tcrig1*,NS; *LOC101882854-001*(NC) ~2.32 F, *LOC101882854-002*(NC)~1.79 F	Less pigmentation
pIDM-A96	3;[*scn8aa*(9, 11^th^ intron, reverse)];[~32.6 kb at 3′ side: *LOC568650* (3)]; [~14.3 kb at 3′ side: *LOC101884758* (4)]	*scn8aa* ~39%; *tmprss12* ~2.76 F	Arrested development (18–21 somite stage)
pIDM-E256	3;[*snx17*(20, 1st intron, reverse)];[*NLRC3-like*(21, 8^th^ exon, reverse)]; [~3.7 kb at 3′ side: *ppp3cca*(5, reverse)]	*snx17* ~73%; *NLRC3-like* ~74%	Arrested development (15–26 somite stage)
pIDM-A199	2;[*si:ch211-220f12*.*4*(18, 1st intron, reverse)];[*ogdhb*(10, ~5.5 kb upstream of 1st exon, forward)]	*prpf18*~2.54 F; *ogdhb*~5.17 F	Curved body with severe cell death
pIDM-A31	3;[*LOC101885416*(4, 3rd intron, reverse)]; [*lox12a*(10, 3rd intron, reverse)]; [~67.7 kb at 3′side: *LOC100331306* (13, forward)]	*lox12a* ~17.6%	Non-specific phenotypes^e^
pIDM-E43	3;[*tbx1*(5,6^th^ intron, reverse)]; [*ablim3*(14, 2^nd^ intron, reverse)]; [*dapk1*(5,6^th^ intron, forward)]	*tbx1* ~40%; *ablim3* ~62%; *dakp1*, NS	Non-specific phenotypes
pIDM-E55	2;[*shisa-7-like*(16, 3^th^ exon, reverse)];[*efnb1*(5,1st intron, forward)]	*shisa-7-like* ~44%; *efnb1*,NS	Non-specific phenotypes
pIDM-E6	7;[*ebag9*(16,1st intron, forward)];[*matn4*(6,1st intron, reverse)]; [*prdm5*(23, 13^th^ intron, reverse)]; [*LOC101883267*(13, ~500 bp upstream of 1st exon, forward)]; [*ube2nb*(4,1st intron, forward)]; [*tbc1d8*(9, 7^th^ intron, reverse)];[*ifnphi3*(3,2^nd^ intron, reverse)]	*ebag9*~3.31 F; *matn4*~55%; *prdm5*~55%; *ube2nb*,NS; *tbc1d8* ~68%; *ifnphi3*, NS	Non-specific phenotypes

^a^Relative level of mRNA: RNA remaining in heterozygous pIDM transgenic embryos treated with Dox relative to wild-type transcript levels; ^b^F: fold; ^c^FDD, fin developmental defects; ^d^DFR, defects in fin regeneration; ^e^non-specific phenotypes: no-midbrain-hindbrain boundaries, lack normal brain ventricles, defects in eye development and heart edema; NC, non-coding RNA; N.D, no data; NS, no significance; pIDM-A: *β-actin* promoter was used to drive rtTA expression; pIDM-E: *elf1a* promoter was used to drive rtTA expression.

### Dox-induced down-regulation of entrapped genes causes abnormal embryonic development in six pIDM mutants

**Figure 2:**
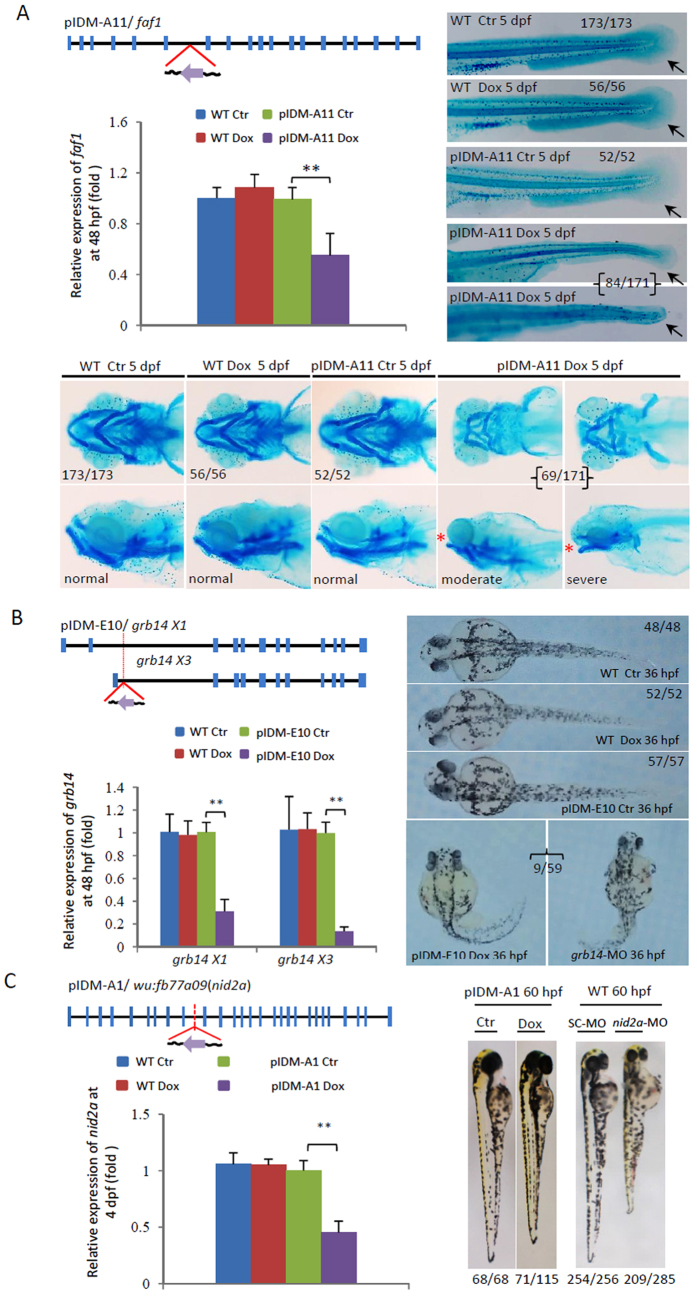
Dox-dependent down-regulation of genes in three example mutants causes abnormal embryonic development. (**A**) Line pIDM-A11. pIDM-A, a *β-act* promoter was used to drive *rtTA* and *Egfp* genes. Diagram showing the position and orientation of pIDM in the *faf1* genomic DNA. Black line, intron or intergenic DNA; Blue vertical bar, exon; Purple arrow, direction of Tet-on promoter; Red lines, position of the insertion. Total RNA was extracted at 48 hpf. The relative expression of the *faf1* transcript was analyzed with qRT-PCR. *β-actin* was used to normalize the total RNA. The embryos treated and untreated with Dox (Ctr) were sampled at 5 dpf and subsequently subjected to alcian-blue-staining for fin (Top right panel) and pharyngeal cartilage (Bottom panel). Black arrow, position of caudal fin; Red star, ‘open-mouse’ phenotype. (**B**) Line pIDM-E10. pIDM-E, an *elf1a* promoter was used to drive *rtTA* and *Egfp* genes. Diagram of the position and orientation of pIDM in the *grb14* genomic DNA (including two isoforms *grb14X1* and *X*3). The relative expression level of *grb*1*4X*1 and *X3* transcripts was analyzed by qRT-PCR with specific primers at 48 hpf. Pictures of WT and mutant embryos with different treatments at 36 hpf as indicated, noting that *grb14*-MO morphants had similar defects as that in pIDM-E10 transgenic embryos upon Dox induction. (**C**) Line pIDM-A1. Diagram of the position and orientation of pIDM in the *wu:fb77a09*(*nid2a*) genomic DNA. The relative expression level of the *nid2a* transcript was analyzed at 4 dpf. Pictures of WT and mutant embryos with differing treatments at 60 hpf as indicated. In A, B and C, representative embryos are shown, the number of embryos showing the displayed phenotype versus total embryos examined are provided in the corresponding panels. All statistically significant differences between samples were assessed with the independent-samples *T*-test (*P < 0.05, **P < 0.01, ***P < 0.001).

We first characterized 6 mutant lines with single-insertions, in which the entrapped gene was down-regulated upon Dox treatment (Table 1). The first mutant, pIDM-A11, displayed a Dox-dependent pericardial edema phenotype in F_1_ embryos (Fig. S4A) and carried an insertion in the 6^th^ intron of the *fas associated factor* 1 (*faf1*) gene with the tet-on promoter oriented in the opposite direction of *faf1* transcription (Fig. 2A). Quantitative reverse transcription-PCR (qRT-PCR) analysis showed that the expression of *faf1* was down-regulated to 50% of normal levels of *faf1* transcripts upon Dox-treatment (Fig. 2A). This was confirmed with whole-mount *in situ* hybridization (WISH) (Fig. S4B). Anti-sense RNA of *faf1* was only detected in the pIDM-A11 embryos upon Dox-treatment (Fig. S4C), suggesting that the down-regulation of *faf1* is mediated by the anti-sense transcript. The *faf1* is highly expressed in the cartilage during embryonic development. A previous study has shown that sporadic cleft palate only (CPO) human patients have significantly decreased expression of *FAF1* ^22^. Knockdown of zebrafish *faf1* with specific morpholino also leads to pharyngeal cartilage defects and jaw abnormality^22^. In addition to having pericardial edema phenotype, pIDM-A11 F_2_ embryos also had abnormal cartilage phenotypes upon Dox-treatment (Fig. 2A; lower panels), which was similar to those in *faf1* morphants. The mutant embryos also had defects in fin development (Fig. 2A; upper right panels).

The *δ-sgcd* was identified from line pIDM-A200. The insertion was in the 2nd intron of both of the two *sgcd* splicing isoforms (*sgcd* and *sgcdX1*) with the Tet-On promoter oriented in the opposite direction of *sgcd* transcription (Fig. S5A). The total expression of the two isoforms in mutants decreased to about 40% due to Dox-induced expression of its anti-sense RNA (Fig. S5B,D). In previous studies, *sgcd* has been linked to limb girdle muscular dystrophy (LGMD) in humans^23^. The *sgcd* zebrafish morphants display abnormal skeletal muscular development, pericardial edema, hypoplastic head and runtish trunk phenotypes^24, 25^. Such phenotypes were also found in 30% of pIDM-A200 mutant embryos upon Dox-treatment (Fig. S5C).

Regarding *adaptor-related protein complex* 2 *beta* 1 *subunit* (*ap2b1*), it has been reported that knockdown of *ap2a1*with a translation-blocking morpholino, an endocytic clathrin-coat component, produces severe phenotypes such as early patterning and axis formation abnormalities, arrested development and many morphants with the phenotypes do not survive to 24 hpf. Surviving morphants at 24 hpf show dysmorphic bulging of the yolk sac such as: extruded-yolk and others^26^. In the pIDM-A17 mutant, *ap2b1* was down-regulated to 40% by Dox-induced expression of its anti-sense RNA (Fig. S6A,B,D). The development of mutant embryos arrested at early stage and the abnormal embryos also displayed an extruded-yolk phenotype upon Dox treatment (Fig. S6C).

The *growth factor receptor-bound protein* 14 *(grb*1*4)*, facilitating avid binding to activate insulin receptor tyrosine kinases, has multiple isoforms in zebrafish. The insertion was located in the 2nd intron of *grb*1*4X1* and the 1st intron of *grb14X3* in line pIDM-E10 with the Tet-On promoter oriented in the opposite direction of *grb14* transcription (Fig. 2B). Dox treatment down-regulated the expression of both isoforms to 30% and 10%, respectively, accompanying with the induction of their antisense RNA (Figs 2B and S7B). The WISH results also showed that the total expression of the two isoforms was reduced upon Dox-treatment (Fig. S7A). pIDM-E10 embryos displayed abnormal development with curved body and small head after Dox induction. This was confirmed with a *grb14* morpholino (*grb14*-MO) designed to block the splicing of both of *grb14* isoforms (Figs 2B and S7C).

The *wu:fb77a09* (*nidogen*2*a*, *or nid*2*a*) encodes a basement membrane protein. The expression of *nid2a* in line pIDM-A1 was decreased to 40% by Dox-induced expression of its antisense RNA (Figs 2C and S8A). The mutant embryos displayed shorter body length than WT. The phenotype was confirmed with a *nid2a* morpholino (*nid2a*-MO) (Figs 2C and S8B). Although in previous studies the loss of either *Nid2* or its family member *Nid1* in mice had no effect on basement membrane formation and organ development^27, 28^, a deficiency of both *Nidogenes* resulted in perinatal lethality and abnormal development of lung and heart, and with limbs displaying syndactyly, and body of smaller size due to defects in basement membrane assembly^29, 30^.

Surprisingly, in line pIDM-E3, the insertion was at about 1 kb upstream of *phosphate cytidylyltransferase 1*, *choline*, *alpha a* (*pcyt1aa*) where the Tet-On promoter was oriented in the same direction of *pcyt1aa* transcription (Fig. S9A). However, the expression of *pcyt1aa* was down-regulated to 30% upon Dox-treatment rather than up-regulated as expected (Fig. S9B). The possible explanation for this phenomenon is that the activation of Tet-On system may interfere with a cis-regulatory element of *pcyt1aa* transcription. The mutants showed smaller eyes with less pigmentation and evident brain hemorrhage (Fig. S9C), which was confirmed by a splicing morpholino *pcyt1aa*-MO (Fig. S9E,F). In humans, a mutation of *pcyt1aa* (encoding a key enzyme in the phosphatidylcholine biosynthesis pathway) was found to result in spondylometaphyseal dysplasia with cone-rod dystrophy (SMD-CRD)^31^.

Previous studies showed that double-strand RNA (dsRNA) injection causes nonspecific developmental defects in zebrafish embryos^32, 33^. To rule out the possibility that the developmental defects in the pIDM mutant lines producing gene specific antisense RNA are due to nonspecific effects of dsRNA, we generated six dsRNAs including *Egfp* (a nonspecific gene control), *nid2a*, *grb14*, *faf1*, *sgcd* and *ap2b1*. Most of the embryos (around 80%) injected with any of these dsRNAs at 10 pg per embryo (10 pg/PE, the lowest concentration leading to abnormal embryos) had general growth arrest before or around the epiboly stage (from 40% to 50%) or displayed nonspecific growth defects with abnormal curved body at 24 hpf (from 25% to 35%) (Fig. S10). The nonspecific growth defects in the dsRNA-injected embryos are similar to those described in the previous studies^32^ but are different from the phenotypes in our gene-specific knockdown mutants.

To investigate whether the down-regulation of the entrapped gene expression was due to RNA interference mediated by the gene specific anti-sense transcript, we replaced *HA-DsRed* of *pIDM:HA-DsRed* vector with the anti-sense of *faf1* or *grb14* coding region cDNA to generate two zebrafish transgenic lines: pIDM-anti-*faf1* and pIDM-anti-*grb14* (Fig. S11A). RT-PCR results showed that the anti-sense RNA of *faf1* and *grb14* was only detected in the respective transgenic embryos upon the Dox-treatment (Fig. S11B). However, the expression of the target gene either *faf1* or *grb14* was not interrupted by the Dox treatment in the transgenic embryos (Fig. S11C), and the transgenic embryos developed normally upon the Dox treatment (Fig. S11D,E), which are different from those in the two gene-entrapped transgenic lines (Fig. 2A,B). The results suggested that the down-regulation of the entrapped gene mediated with the anti-sense transcript in pIDM lines, might be through locally interrupting mRNA transcription or splicing or stability, rather than through RNA interference pathway remotely.

In addition, we generated one more *nid2a* mutant allele with a 7-bp deletion and a 27-bp insertion in exon 2 using the CRISPR/Cas9 technique (Fig. S12A). Not surprisingly, the nid2a^*−/−*^ homozygous mutant embryos also displayed shorter body length at 4 dpf as those in the pIDM-A1 mutant embryos under the Dox treatment (Fig. S12B,C). Taking together, the data demonstrated that abnormal phenotypes in the Dox treated pIDM mutants were resulted from the specific gene knockdown.

### Dox-induced up-regulation of entrapped genes leads to abnormal embryonic development in two representative pIDM mutants

**Figure 3:**
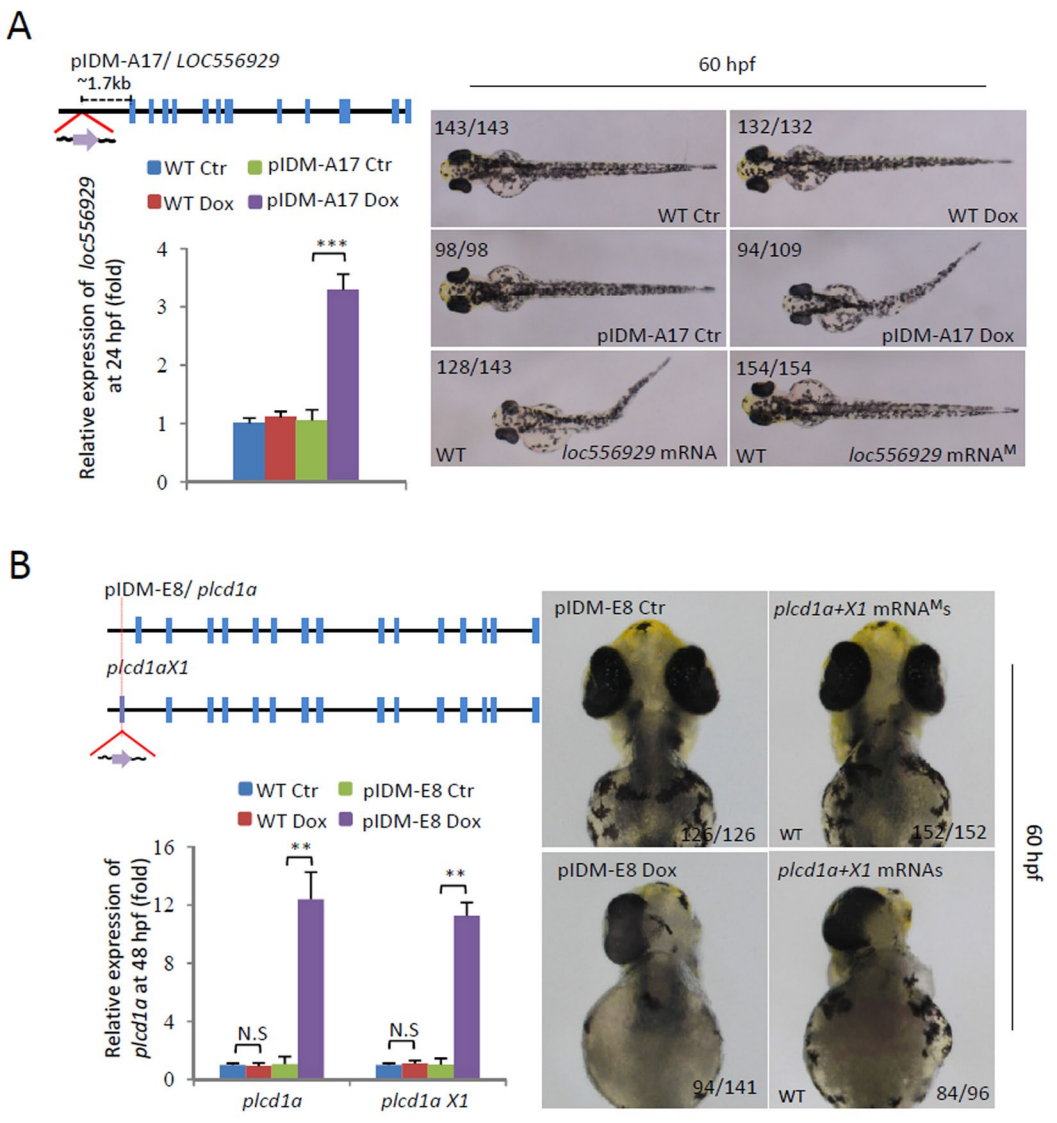
Dox-dependent up-regulation of genes in two example mutants causes abnormal embryonic development. (**A**) Line pIDM-A17. Upper left panel: Diagram showing the position and orientation of pIDM in *loc*5*569*2*9* genomic DNA. Bottom left panel: The relative expression level of the *loc556929* transcript upon Dox-treatment was analyzed at 24 hpf. Right panel: Pictures of WT and mutant embryos with different treatments at 60hpf as indicated, noting that over-expression of wild-type *loc556929* mRNA, but not mutant *loc556929* mRNA^M^, phenocopied embryonic defects in pIDM-A17 transgenic embryos after Dox induction. (**B**) Line pIDM-E8. Upper left panel: Diagram showing the position and orientation of pIDM in the *plcd1a* genomic DNA (including two isoforms *plcd1a* and*X1*). Bottom left panel: The relative expression levels of *plcd1a* and *X1* transcripts upon Dox-treatment was analyzed with specific primers at 48 hpf. Right panel: Pictures of WT and mutant embryos with differing treatments at 60 hpf as indicated, noting that over-expression of wild-type *plcd1a* + *X1* mRNAs, but not *plcd1a* + *X1* mRNA^M^s, phenocopied embryonic defects including a single eye in pIDM-E8 transgenic embryos after Dox treatment. In A and B, representative embryos are shown, the number of embryos showing the displayed phenotype versus total embryos examined are provided in the corresponding panels.

Up-regulation of entrapped gene expression was found in two lines, pIDM-A17 and pIDM-E8, both harboring a single-insertion. In line pIDM-A17, the insertion was at 1.7 kb upstream of the transcription start site of the novel gene *LOC556929* with the Tet-On promoter in the same direction of *LOC556929* transcription (Fig. 3A; left upper panel). The expression of *LOC556929* was increased to 3.3 fold upon Dox-treatment, as compared to that of the untreated control (Fig. 3A; left lower panel). Overexpression of *LOC556929* resulted in abnormal development with curved body in this pIDM mutant (Fig. 3A). Consistently, a similar phenotype was observed in the WT embryos injected with *LOC556929* mRNA, but not in the embryos injected with *LOC556929* mutant mRNA carrying an early stop codon (Fig. 3A; right panel).

In line pIDM-E8, the insertion was at 1035 bp upstream of the transcription start site of the *phospholipase C*, delta 1a(*plcd1a*) gene and in the first exon (75 bp upstream of the start codon ATG) of its isoform *plcd1aX1* (Fig. 3B; left upper panel) with the Tet-On promoter in the same direction of *plcd1a* transcription. The expression of both isoforms in the mutants was increased more than 10 fold upon Dox-treatment (Fig. 3B; left lower panel). In the pIDM-E8 mutant, the upregulated expression of the two isoforms resulted in abnormal embryogenesis with delayed eye development, small brain and curved body at 36 hpf (Fig. S13). Most of mutant embryos developed with only one-eye at 60 hpf upon Dox treatment (Fig. 3B). This was confirmed by co-injection of *plcd1a* mRNA with its *X1* isoform mRNA in WT embryos, but not by the mutant mRNAs with an early stop codon (Figs 3B and S13). These data suggest that this pIDM system can also lead to identifying gain-of-function mutants that are controllable by Dox induction.

### Dox-dependent embryonic developmental mutants with multiple insertions

Additional analyses revealed that 16 Dox-dependent embryonic developmental mutant lines harbored multiple insertions (2 to 7) (Table 1). A total of 46 insertion sites in these 16 mutant lines were determined with linker mediated-PCR (LM-PCR), in which 36 insertions were in intragenic regions, 7 insertions were in intergenic regions, and 3 insertions were not determined. The 36 intragenic insertions were predicted to affect 54 genes, including 42 protein-coding genes and 12 non-coding genes. Of the 42 coding genes, 37 were known genes that were studied in either zebrafish or other organisms, and 5 were novel genes with unknown functions. Among these 42 coding genes, the expression of 19 was down-regulated, the expression of 13 was upregulated, the C-terminal part of two genes was ectopically expressed, the expression of 5 genes remained unchanged, and the expression of 3 genes was not detectable (Table 1). Among the 16 mutant lines, 4 of the 12 non-coding genes were down-regulated, 4 were ectopically expressed, 1 remained unchanged, and 3 were not detectable (Table 1). Future studies are warranted if the abnormal development was due to the perturbation of these non-coding genes.

The phenotypes of all 16 mutant lines are presented in Fig. S14. Most lines with multiple insertions showed Dox-dependent specific phenotypes. These include shorter body length in pIDM-A28 (Fig. S14K), curved body and smaller head in pIDM-E19 (Fig. S14G), shorter and thicker yolk sac extension in pIDM-E54 (Fig. S14F), no evident pericardium in pIDM-E14 (Fig. S14E), deformity with severe cell death in pIDM-A199 (Fig. S14H), and epidermal blisters in pIDM-A33 (Fig. S14B). However, 4 of the 16 mutant lines including pIDM-E55, -A31, -E43 and -E6, displayed non-specific phenotypes such as: no-midbrain-hindbrain boundaries, lack of normal brain ventricles, defects in eye development and heart edema (Fig. S15A–D), which were quite similar to those of the MZ*dicer* mutant^34^. The *dicer* mutant phenotypes were also observed in the embryos injected with the siRNAs of many genes^32, 33, 35^. These results suggest that the non-specific phenotypes might be caused by excessive antisense-RNAs and/or sense RNAs derived from different entrapped genes in those mutants with multiple insertions.

### The concept and efficiency of this inducible dominant mutagenesis is validated by a more focused genetic screen for cardiovascular mutants

**Figure 4:**
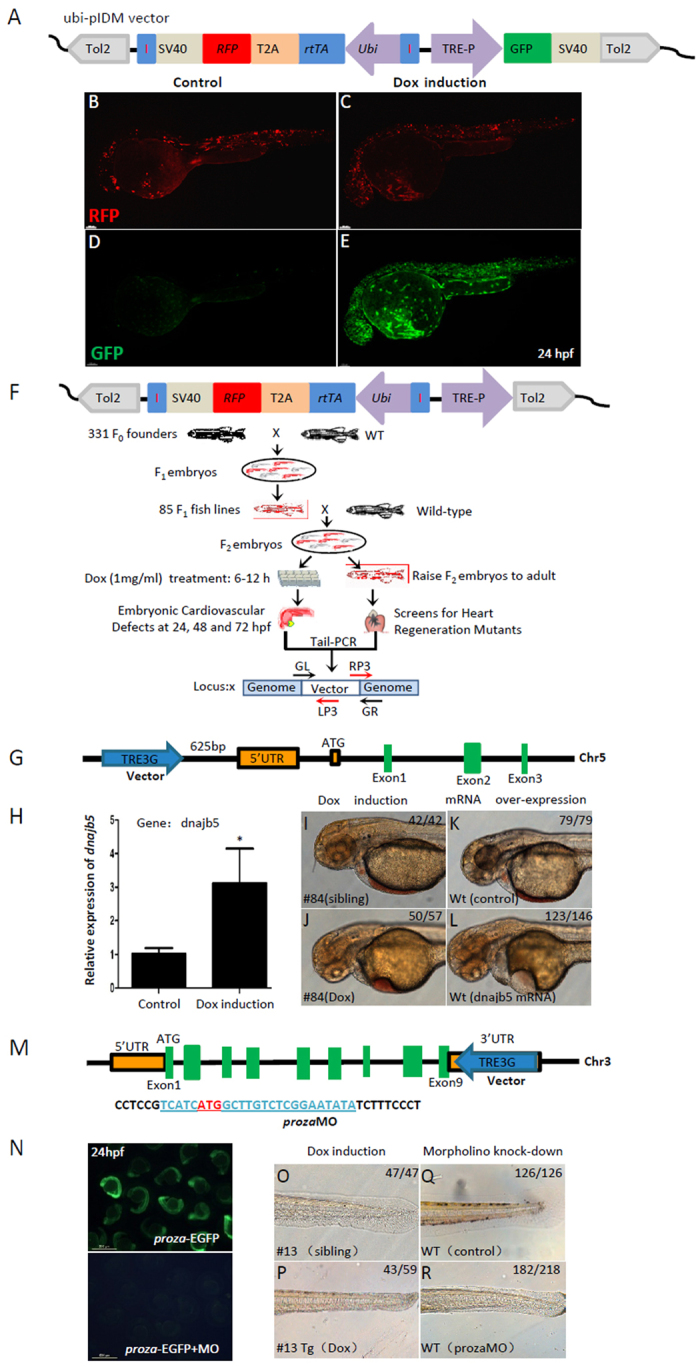
The inducible dominant mutagenesis is validated in a more focused genetic screen for cardiovascular mutants. (**A**) A modified pIDM vector (ubi-pIDM) for testing Dox-induced GFP expression. GFP is located downstream to TRE3G promoter, rtTA and transgenic reporter RFP are driven by the ubi promoter, of which rtTA and RFP are linked by T2A, and the ubi-rtTA-T2A-RFP cassette is flanked by a pair of insulators. (**B–E**) Transgenic reporter RFP of F_1_ embryos were expressed in both control (**B**) and Dox-induced (**C**) embryos. GFP was hardly expressed in control group (**D**) but highly induced by Dox (**E**). (**F**) Schematic of the screen strategy for cardiovascular mutants. F_0_ founders were crossed with WT to establish F_1_ transgenic zebrafish. The F_1_ transgenic fish with RFP was crossed with WT to get F_2_ embryos. The F_2_ embryos were divided into two groups, one treated with Dox at 6–12 hpf for screening cardiovascular defects, and another one for raising F_2_ embryos into adults. (**G**) The ubi-pIDM vector insertion is located at 625 bp upstream to the 5 ‘UTR of *dnajb5* in #84 line. (**H**) RT-PCR showed that *dnajb5* mRNA was 3 times more after Dox induction. *p < 0.05. (**I–L**) #84Tg had cardiovascular defects (**J**) compared with WT siblings (**I**) at 48 hpf after Dox induction. Embryos injected with *dnajb5* mRNA (0.1ng) (**L**) mimicked the phenotypes of Dox treated #84Tg embryos. Lower right numbers show phenotypical embryos out of total embryos analyzed. (**M**–**R**) Cartoon shows the ubi-pIDM vector position in the 3 ‘UTR of *proza* in Chr. 3 in #13 line. The *proza* 5′UTR sequence was used to drive EGFP expression, the start code ATG of *proza* is in red and underlined, and the prozaMO sequence is underlined (**M**). The proza:EGFP reporter was expressed in control embryos (upper panel) that was inhibited by prozaMO (4 ng) (lower panel) at 24 hpf. (**O**–**R**) #13 transgenic embryos (#13Tg) showed no blood circulation and abnormal tail development (**P**) compared with #13 control siblings (**O**) after Dox induction. Morphant embryos with 4 ng prozaMO showed developmental defects (**R**) similar to that in #13Tg embryos (**P**) at 48 hpf.

To further improve the efficiency of this pIDM-based mutagenesis of the zebrafish genome, we replaced the β-actin promoter-rtTA-IRES-EGFP-SV40 poly-A cassette (Fig. 1A) with the ubi-promoter-rtTA-T2A-RFP-SV40 poly-A cassette, generating a new ubi-pIDM vector (Fig. 4A). The zebrafish ubiquitin promoter was reported to drive better ubiquitous transgene expression from embryos to adult organs than that by the β-actin promoter^36^, and the self-cleaving 2 A peptide (T2A) was shown to be a better candidate to replace IRES because of its small size and high cleavage efficiency between genes upstream and downstream of the 2A peptide^37^. We first tested whether the ubi-pIDM vector is tightly controlled by Dox induction, so we included the reporter gene GFP under control of the Tet-On promoter (TRE3G) to form the ubi-pIDM-GFP vector (Fig. 4A). As expected, we found evident expression of GFP after Dox induction compared with that by DMSO control while RFP was expressed in either the presence or absence of Dox (Fig. 4B–E), suggesting the Tet-On promoter is tightly controlled by Dox induction.

We then used ubi-pIDM vector to carry out a more focused genetic screen for cardiovascular mutants (Fig. 4F) that were similar to the one described above (Fig. 1). Out of 331 RFP-positive F_0_ transgenic founders, we isolated 85 F_1_ founder lines, representing about 26% germline-transmission and an averaged 3 insertions from each transgenic line. By crossing transgenic F_1_ founders with wild-type zebrafish, we collected F_2_ transgenic embryos, which contained mixed transgenic embryos and wild-type siblings. We then divided them into two groups, one subjected to Dox induction for mutant screens and the other raised to adults. We applied Dox treatment from 6 to 12 hpf, and scored cardiovascular phenotypes at 24, 48, and 72 hpf. These efforts led to isolation of 14 transgenic lines that had Dox-induced cardiovascular and/or circulation defects. Since some of the 14 transgenic lines contained more than one insertions, we validated this phenotypic screen with F_3_ transgenic embryos by segregating transgenic insertions, and subsequently confirmed 10 single-insertion transgenic lines to have identical phenotypes to those in their F_2_ transgenic embryos (Table 2). By using Tail-PCR^38^, we cloned all insertions from each of the 7 transgenic lines, and determined the responsible genes by performing genotype-phenotype correlation analyses. On the other hand, we were not able to determine the affected genes from 3 transgenic lines. The transgenic line names, phenotypes, responsible genes, insertion positions, and chromosomal locations are summarized in Table 2 and Fig. S16A,B. WISH assays revealed that *Dnajb5*, *proza*, *St6galnac1*.*2*, and *sps2* were expressed in the heart (Fig. S16C).

**Table 2:** Information of the ubi-pIDM mutants with blood circulation and cardiovascular defects.

Line^#^	Phonotypic defects	Responsible genes	Transcripts	Gene types	Insertion position	Location
6	heart, circulation, tail	*si:ch73-212p10*.*3*	antisense	non-coding gene	1/4 intron	Chr: 7
8	heart, circulation	*st6galnac1*.*2*	sense	coding gene	5′UTR	Chr: 12
13	circulation, tail	*proza*	antisense	coding gene	3′UTR	Chr: 3
23	heart, circulation	*bahcc1*	sense	coding gene	intron12/28	Chr: 12
34	heart, circulation, tail	*undetermined*	undetermined	undetermined	undetermined	undetermined
37	heart, circulation	*sps2*	antisense	coding gene	5′UTR	Chr:23
43	heart, circulation	*cacna2d4*	antisense	coding gene	16/37exon	Chr:25
54	heart, circulation, tail	*undetermined*	undetermined	undetermined	undetermined	undetermined
76	heart, circulation, tail	*undetermined*	undetermined	undetermined	undetermined	undetermined
84	heart, circulation	*dnajb5*	sense	intergenic region	intergenic region	Chr: 5

The phenotypic screen identified 10 mutant lines based on blood circulation and cardiovascular abnormalities of transgenic embryos after Dox induction. Seven single-insertion positions and responsible genes were determined, including 1 non-coding gene and 6 coding genes, of which 3 lines are predicted to generate sense transcripts and 4 lines for antisense transcripts. The single-insertion sites of 3 transgenic lines are not determined.

To better understand how this new ubi-pIDM vector works, we chose to systematically characterize two representative transgenic lines, ubi-pIDM#84 and #13. In ubi-pIDM#84 line, we found that the vector inserted at 625 bp upstream to 5′UTR of *dnajb5* gene and the Tet-On promoter had the same orientation as dnajb5 gene transcription (Fig. 4G). RT-PCR confirmed that *dnajb5* increased in transgenic embryos after Dox induction (Fig. 4H). Consistently, over-expression of dnajb5 mRNA in wild-type embryos resulted pericardial edema and abnormal heart development as that in ubi-pIDM#84 transgenic embryos in the presence of Dox (Fig. 4I–L). In ubi-pIDM#13, we found that the insertion occurred in the 3′UTR of proza gene and the Tet-On promoter had opposite direction as that of proza gene transcription (Fig. 4M). We did not find evident down-regulation of proza mRNA in this transgenic embryos after Dox induction (data not shown), but we found that proza morphants had similar circulation and tail defects as those in this transgenic embryos after Dox induction (Fig. 4O–R). The efficiency of proza ATG morpholino was determined based on its suppression of EGFP expression driven by the proza 5′UTR (Fig. 4N). Together, these data further substantiate the notion that the ubi-pIDM, like the above pIDM, provides an effective genetic tool for isolation of inducible cardiovascular mutants, characteristic of either gain-of-function or loss-of-function mutations.

Here, we should note that two transgenic systems (β-act/elf1a-pIDM and ubi-pIDM) were generated by two different groups. It would not be appropriate to compare the transgenic and phenotypic mutant frequencies between two constructs, since there were a number of differences between two screens such as: ubiquitous promoters, reporter genes, mutant phenotypes and so on. Nevertheless, the two similar but different transgenic pIDM systems had comparable efficiency in isolating a number of zebrafish mutants for studying gene function in embryos and adults.

### This inducible pIDM system opens a novel avenue for identification of adult caudal fin regeneration mutants

**Figure 5:**
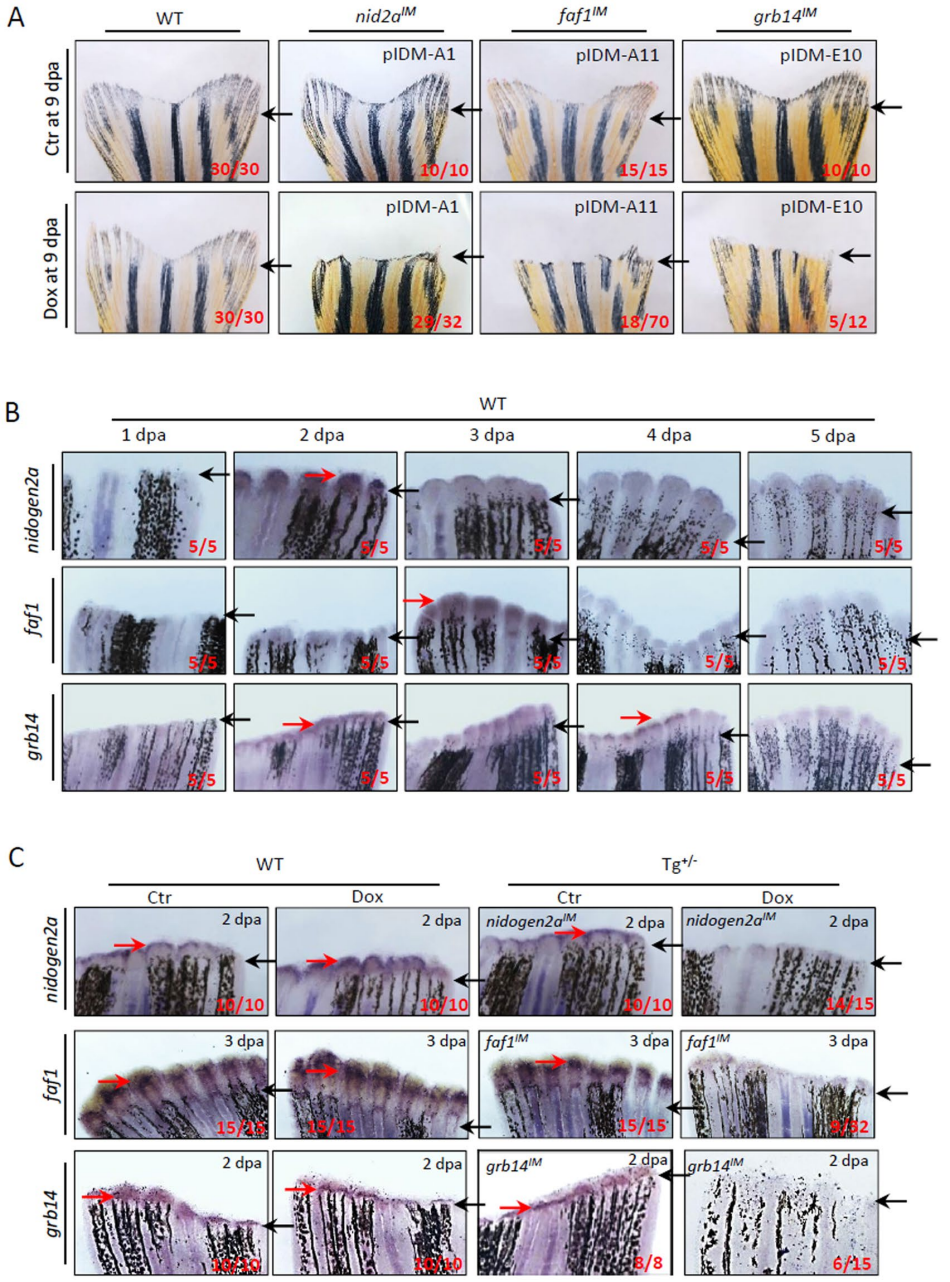
Dox-induced down-regulation of genes in three single-inserted example mutants inhibits adult caudal fin regeneration. (**A**) Adult caudal fin failed to regenerate after Dox-treatment in three mutant lines pIDM-A1 (*nid2a*), pIDM (*faf1*), and pIDM (*grb14*) at 9 days post amputation (dpa). Black arrow, cutting site. (**B**) All three genes were induced in wild-type caudal fins after amputation. The WT caudal fin was sampled at indicated time points after amputation and subjected to a WISH assay with the corresponding anti-sense RNA probes as indicated. Black arrow, cutting site; Red arrow, positive signal. (**C**) Dox-induced down-regulation of the entrapped genes is found in respective transgenic mutant lines compared with wild-type fins with or without Dox or control transgenic fins without Dox by WISH assay. The amputated caudal fin was sampled at 2 or 3 dpa. Black arrow, cutting site; Red arrow, positive signal. In a–c, representative fish are shown, the number of fish showing the displayed phenotype versus total fish examined are provided in the corresponding panels.

A key advantage of this mutagenesis system is that it opens an avenue for the screening of genetic dominant mutants at the adult stage. For easy readout of mutant phenotypes at the adult stage, we screened mutants with fin regeneration defects. From 24 Dox-dependent abnormal embryonic mutant lines and 95 pIDM transgenic lines without obvious developmental defects, we identified 4 (out of 24) and 3 (out of 95) lines with impaired caudal fin regeneration at 9 days post amputation (dpa), respectively (Table 1). Three fin regeneration mutants had single-insertion mutations, including pIDM-A1 (*nid2a* ^*IM*^), pIDM-A11 (*faf1* ^*IM*^) and pIDM-E10 (*grb14* ^*IM*^) (Fig. 5A). In WT zebrafish, WISH assays showed that the expression of *nid2a*, *faf1* and *grb14* was obviously up-regulated around the amputation plane at 2, 3 and 2 dpa, respectively, and then down-regulated to the basal level at 5 dpa during fin regeneration (Fig. 5B). In contrast, the up-regulation of the three genes during fin regeneration was abolished in their respective mutants upon Dox-treatment (Fig. 5C). This implicates that the fin regeneration defect was due to the knockdown of these three genes in their respective mutants. Notably, our data is consistent with a previous report that the up-regulation of *nid2a* during zebrafish fin regeneration has been also observed using microarray analysis^39^.

### Segregation of multiple insertions in 3 fin regeneration mutants allows us to determine their affected genes

**Figure 6:**
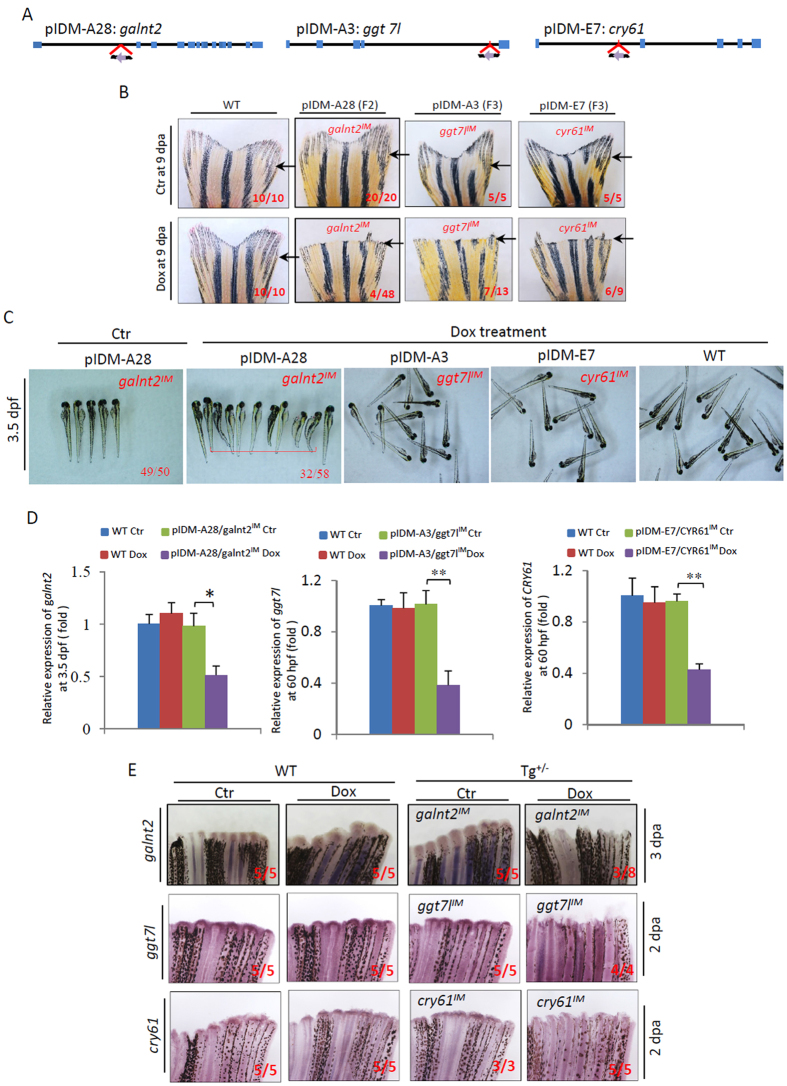
Three single-insertion mutant lines, segregated from multiple insertions, have adult caudal fin regeneration defects after amputation. (**A**) Diagram showing three single-inserted pIDMs in the *galnt2*, *ggt7l* and *cry61*genomic DNAs segregated from lines pIDM-A28, A3 and E7. (**B**) Fin regeneration defects upon Dox-treatment in pIDM-A28 F_2_ mutants with single-insertion *galnt2* ^*IM*^, pIDM-A3 F_3_ mutants with single-insertion *ggt7l* ^*IM*^, and pIDM-E7 F3 mutants with single-insertion *cry61l* ^*IM*^ as indicated. The experiments were performed as described in Fig. 4A. Black arrow, cutting site. (**C**) Images showing WT and mutant embryos of the three single-insertion lines upon Dox treatment at 3.5 dpf as indicated. (**D**) The relative expression level of *galnt2*, *ggt7l* and *cry61* transcripts decreased in segregated mutant embryos at 3.5 dpf upon Dox treatment as indicated. (**E**) Down-regulation of the entrapped gene expression was found in respective segregated mutant lines upon Dox-treatment as indicated time points. The amputated caudal fin was sampled at 2 or 3 dpa. In **B**, **C** and **E**, representative fish are shown, the number of fish showing the displayed phenotype versus total fish examined are provided in the corresponding images.

The remaining 4 lines with Dox-dependent fin regeneration defects, including pIDM-A28 (with shorter body length phenotype at embryonic stages) (Fig. S14K), as well as pIDM-A18, -A3 and -E7, of which all 3 lines displayed no obvious phenotypes at the embryonic stages, belong to multiple-insertion lines (Table 1). To identify the causative mutation, we performed segregation and linkage analysis for each of the 4 multiple-inserted lines (The details of the procedure in Detailed protocol of Supplementary Materials). Using southern blot and gene-specific PCR, we identified three single-inserted genes (*galnt2*, *ggt7l* and *cry61*) responsible for fin regeneration defects from pIDM-A28, A3 and E7 respectively (Figs S17A,B,C and 6A,B). The single insertion respectively located in the 1st intron of *galnt2*, the 4^th^ intron of the *γ-glutamyltransferase7-like* (*ggt7l*) and the 1st intron of *cry61* with the Tet-On promoter oriented in the opposite direction (Fig. 6A). Dox-dependent fin regeneration defective phenotype was observed in all of the segregated three lines (9.2% in pIDM-A28, 54% in pIDM-A3 and 66.7% in pIDM-E7) (Fig. 6B), whereas EGFP positive embryos from these segregated lines did show similar Dox-dependent phenotypes as the embryos from the F_1_ generation (shorter body length in pIDM-A28, no obvious phenotypes in both of pIDM-A3 and E7) (Fig. 6C). The expression of all three genes (*galnt2*, *ggt7l* and *cry61*) was knocked down to about 40% upon Dox-treatment (Fig. 6D). WISH assays showed that the expression of three genes was up-regulated from 2 dpa during WT fin regeneration and the injury-induced expression of the three genes was abolished in the pIDM mutant fish after Dox-treatment (Figs S17D and 6E).

The *galnt2* encoded N-acetylgalactosaminyltransferase2, the enzyme that catalyzes the initial step of mucin type-O glycosylation. Previous studies showed that GALNT2 plays a dual role in mediating cancer cell migration and invasion in different types of cancer cells via modifying O-glycosylation and activity of EGFR^40, 41^. In zebrafish, a microarray study showed that *galnt1* and *galnt6* (family-members of *galnt2*) were upregulated during fin regeneration^39^. The *ggt7l* is a member of γ-glutamyltransferase family. GGT is the first major enzyme of the gamma-glutamyl cycle that regulates the metabolism of the antioxidant glutathione and also plays a role in inflammatory response^42^. Cyr61 (CCN1) belongs to the CCN family of extracellular matrix-associated protein. It has been demonstrated that human CYR61 regulates genes involved in wounding healing and mouse Cyr61 is essential for placenta development and vascular integrity^43, 44^. Our results suggest that these three genes also play a role in organ regeneration.

We failed to segregate into single-insertion from line pIDM-A18, since there were two insertions which were closely linked in the mutant (data not shown).

Taken together, our data demonstrates that this pIDM system is an ideal conditional system for mutant screens in adult zebrafish, presenting unprecedented opportunity of identifying both gain-of-function and loss-of-function mutants for studying adult organ regeneration.

## Discussion

In combining the Tol2 transposon with a Tet-On promoter we have developed a novel inducible system for dominant mutant screening in zebrafish. In this design, the Tol2-trapped genes can be altered by either overexpression or knockdown. Using the Tet-On expression system^45^, we have implemented a true conditional/inducible mutagenesis system. Through Dox treatment, we are able to perturb target gene functions in heterozygous mutants at any developmental stages and thus screen for genetic mutants in either embryonic or adult stages. Together with the advantage of a near-random integration of Tol2 transposon in the vertebrate genome^46, 47^, this system provides an excellent opportunity to study biological processes especially in the adult stage such as organ regeneration, behavior and memory. This differs considerably from the gain-of-function screen in *Drosophila* mediated with P-elements and in zebrafish mediated with MMLV viral vector that are preferentially inserted at the 5′ ends of genes^7–9^. With Tol2 transposon-mediated pIDM screen system, we identified 72 insertions from 37 pIDM mutants. Among these 72 insertions, the expression of 35 entrapped genes with the insertion oppositely located at intragenic region was downregulated and the expression of 14 entrapped genes wth the insertion at the 5′ end was upregulated. Our data suggest that both of gene knockdown and overexpression can be achieved by the pIDM system. To better understand the mechanisms for the anti-sense transcript to downregulate the expression of the entrapped gene, we generated two transgenic lines: pIDM-anti-*faf1* and pIDM-anti-*grb14*. Although the anti-sense of either *faf1* or *grb14* was induced, the expression of the respective target gene was not interrupted and the transgenic embryos developed normally in the two transgenic lines upon the Dox treatment. One of possible explanations for the downregulation of entrapped gene expression in pIDM mutants is that the anti-sense transcript can only locally interrupt the transcription or splicing or stability of the target gene.

Typically, mutations generated with the conventional mutagens including chemicals, ionizing radiation, and gene-trap strategy cause reduction or elimination of gene function. It takes three generations to get homozygous mutants, which is time-consuming and requires a lot of zebrafish facilities^48^. Using the inducible dominant system for mutant screening, we are able to screen genetic mutants in the F_1_ heterozygous background, though transgenic lines with multiple insertions require outcrossing to segregate the responsive insertion.

A few forward genetic screens include conditional or inducible systems. It is worthy of noting that two recent reports have described a conditional *in vivo* gene-trap mutagenesis system in the zebrafish. In one of these, the mutant alleles are able to be reverted in the somatic tissues via Cre recombinase or splice-site-blocking morpholinos^49^. However, in this technique, the reverted mutant alleles cannot be mutated back since the mutagenic core has been completely excised by Cre recombinase. In another report, the gene-trap cassette was flanked with two pair of recombination sites of Cre and Flp. Cre- and Flp-mediated recombination switches the orientation of the gene-trap cassette, permitting conditional rescue in one orientation and conditional knockout in the other^50^. By applying double-recombination, it is applicable to study the function of the target genes at adult stages. However, the mutant needs to cross with two transgenic lines with conditionally expressing either Cre or Flp recombinase. With the inducible mutagenesis system in this work, we can easily interrupt the expression of the target gene by Dox treatment and restore to normal expression by withdrawal of Dox.

In gene-trap and enhancer-trap screening, the expression of the reporter gene is dependent on the promoter of endogenous target genes^47, 51^. In our inducible dominant mutagenesis system for a mutant screen, rtTA and the reporter gene are expressed by a constitutive promoter. It has been well documented in transgenic researches that random transgenic insertions can lead to undesirable effects, such as heterochromatin silencing or position effects^52–55^. Insulators have been successfully used to overcome variegation of transgene and increase expression of transgene^21^. In this case, the addition of a pair of the chicken β-globin insulators in our system not only increased the intensity of EGFP, but also doubled the frequency of transgenic lines with visible EGFP signal.

From our fin regeneration mutants, six genes have been identified. It has been shown that two of them or its family members (*nid2a* and *galnt2*) were upregulated during fin regeneration in a microarray study^56^. The *nid2a* and *galnt2* inducible mutants furnish evidence that base membrane and mucin type-O glycosylation are required for fin regeneration. Other 4 identified genes (*faf1*, *grb14*, *GGT* and *Cyr61*) have not been reported to have linkage to fin regeneration yet. It has been known from literature that *faf1*promotes β-catenin degradation^57^, *grb14* is related to insulin receptor signaling^58^, *GGT* plays a role in inflammatory response^42^ and *Cyr61* is involved in wound response^43, 44^. All of these signal pathways have been documented to play an important role in fin regeneration. Future investigations on these inducible mutants will likely gain novel insights of molecular mechanisms in fin regeneration.

This inducible dominant mutagenesis system for mutant screening developed here is complementary with conventional forward genetics. For developing a next generation of the conditional/inducible dominant screen, the ubiquitous promoter could be replaced with tissue-specific promoters, thus allowing us to perform more focused genetic screens on particular organs or molecular pathways. Transposon-mediated DNA-insertion and chemical-induced Tet-On techniques are not only applied in zebrafish, but also widely used in a variety of vertebrates such as mice and *Xenopus*. So our new system is expected to be applicable in mice and *Xenopus*, not only just for studying organ regeneration, but also for studying behavior and memory.

## Materials and Methods

All vectors and 37 mutants lines (Tables 1 and 2) with identified insertions, are freely available and accessible from the China Zebrafish Resource Center (http://www.zfish.cn).

### Ethics Statement

All animal procedures were performed in full accordance to the requirement by ‘Regulation for the Use of Experimental Animals in Zhejiang Province or Peking University’. This work is specifically approved by the Animal Ethics Committee in the School of Medicine, Zhejiang University (ETHICS CODE Permit NO. ZJU2015-516-15, issued by the Animal Ethics Committee in the School of Medicine, Zhejiang University) and Peking University (zebrafish protocol IMM-XiongJW-3).

### Zebrafish lines and maintenance

The Zebrafish (*Danio rerio*) wild type (WT) AB strain was used in this study. The animals were raised and maintained according to standard procedures described in ZFIN.

### Plasmid construction

The pT2HB-PUHrT62 and pT2-β-*actin* plasmids were gifts from Professor Zongbin Cui in the Institute of Hydrobiology, Chinese Academy of Sciences (unpublished); T2KXIG^47^ and pTol2mini (pDB739)^59^ vectors were kindly provided by Professor Koichi Kawakami and Professor Stephen C Ekker respectively.

The *rtTA* DNA fragment was amplified from the pT2HB-PUHrT62 plasmid. The amplified *rtTA* DNA fragment was cloned into a pIRES_2_-EGFP plasmid (Clontech) to generate the prtTA-IRES2-EGFP construct. TRE3G promoter^60^ was synthesized by the Biosune Company.

The core element of the chicken β-globin insulator was synthesized by the Biosune Company. *Construction of ubi-pIDM vector:* The ubi-promoter-rtTA-T2A-RFP-SV40 poly-A cassette was used to replace the *β-actin* promoter-rtTA-IRES-EGFP-SV40 poly-A cassette to form ubi-pIDM vector. The ubi promoter was from the Tg(ubi:loxP-DsRed-STOP-loxP-EGFP) plasmid clone as previously reported^61^, and the T2A peptide was described previously^37^.

The sequences of all primers are provided in Supplementary Table 1. The details of the construction are provided in Detailed protocol of Supplementary Materials.

### Generation of transgenic fish

*Tol2* mRNA was synthesized with T3 RNA polymerase from a pT3TS-Tol2 (pDB600)^59^ plasmid generated in Professor Stephen C Ekker’s lab.

Zebrafish fertilized eggs at the one-cell stage were injected with 1 nano-liter (nl) of a mixture containing 40 pico-gram (pg) circular plasmid DNA of pIDM and 40 pg Tol2 transposase mRNA.

To generate the pIDM:HA-DsRed transgenic line, 100 pg pIDM:HA-DsRed plasmid linearized with NotI was injected into fertilized eggs at the one-cell stage.

### Doxycycline treatment

To reduce side-effects of Doxycycline (Dox) treatment, zebrafish embryos were treated with 30 μg/ml Dox at 12 hpf and then incubated in the dark at 29 °C. Adult fish at 3-4 months old were treated with 60 μg/ml Dox and also incubated in the dark at 29 °C.

### Fin amputation

Dox-treated adult fish at 15 dpt were firstly anaesthetized with tricaine and then half of the caudal fin was removed with scissors. The amputated fish were incubated in the dark in water containing Dox at 29 °C.

### RNA analysis and qRT-PCR

Total RNA was isolated using a Trizol reagent (AidLab) according to the Manufacturer’s protocol. For quantitative real-time reverse transcription PCR (qRT-PCR), total RNA was treated with DNaseI prior to reverse transcription and purified with an RNA clean kit (AidLab). Real-time PCR was performed in a CFX96^TM^ Real-Time System (Bio-Rad) using a C1000 Thermal Cycle (Bio-Rad) according to the manufacturer’s instructions. Total RNA was normalized with *β-actin* or 18S *rRNA*. Two pairs of primers were respectively used to detect the transcript levels of *efnb1*, *dapk1* and *LOC445149* genes for the 5′ region and 3′ region flanking the TRE promoter. Statistics were obtained from three repeat experiments. The primer sequences of analyzed genes are listed in Supplemental Table 1.

For analysis of the anti-sense transcripts of target genes in the Dox treated mutants, DNaseI digested RNA was used to generate the first strand sense-cDNA with the gene specific cDNA forward primer of the target genes. A pair of primers was designed to amplify the region of the target gene located downstream of the pIDM Tet-on promoter. The amplified DNA fragments were subjected to gel electrophoresis. The cycle number of qRT-PCR was performed as follows: 30 cycles (*faf1*), 30 cycles (*grb14*), 33 cycles (*nidogen2a*), 31 cycles (*sgcd*), 31 cycles (*ap2b1*), and 31 cycles (*galnt2*).

For analysis of different splicing transcripts of target genes in the specific morpholino injected embryos, a pair of primers were designed to amplify two joined exons of the target gene, in which one of the slicing sites is blocked by the morpholino. The sequences of all of the primers are provided in Supplementary Table 1.

### Whole mount *in situ* hybridization (WISH)

The use of WISH for embryos has been described previously^62^. The analysis of WISH for caudal fins was performed according to a previous study^63^. The region of 1-400 *faf1*, 25-555 *pcyt1aa*, 124-522 *grb14*, 582-1002 *nidogen2a*, 3045-3733 *galnt2*, 92-519 *ggt7l*, 680-1185*cyr61*, *dnajb5*, *proza*, *st6galnac1*.*2* and *sps2* cDNA was cloned into a pGEMT-Easy plasmid using the gene-specific primer pairs as listed in Table S1.

### Morpholinos and mRNA injection

The *grb14-*MO^spl^ (5′-ACGCGCACACACTTACAAGAGGTTT-3′), the *pcyt1aa*-MO^spl^ (5′-AGACCCTGAGGTGTAAGAAGCATCA-3′) and the *nidogen2a*-MO^spl^ (5′-CCTCAAAGCTGTTTTCTTACCCGAT-3′) were designed to specifically target the splice junction between exon 3 and intron 3 of *grb14*, the splice junction between intron 4 and exon 5 of *pcyt1aa*, and the splice junction between exon 2 and intron 2 of *nidogen2a*, respectively. The morpholinos were supplied by Gene Tools. One nl of 0.5 mM *grb14-*MO^spl^ or *nidogen2a*-MO^spl^ or 1 mM *pcyt1aa*-MO^spl^ was injected into the yolk of one-cell-stage embryos. Human *β-globin* antisense morpholino (5′-CCTCTTACCTCAGTTACAATTT-3′) was used as the standard control.

The *proza*-MO (5′-TCATCATGGCTTGTCTCGGAATATA-3′) was designed to specifically target the ATG. 2 nl of 0.5 mM proza-MO was injected into the yolk of one-cell-stage embryos.

For mRNA injection, 400 pg of *in vitro* transcribed mRNA including *Loc556929*, *plcd1a*, *plcd1a X1*, *dnajb5*, *Loc556929* ^*stop*^[31^th^amino acid (aa), CAG (Gln) to TAG (stop codon)], *plcd1a* ^*stop*^[54^th^ aa, AAG (lys) to TAG], *plcd1a X1* ^*stop*^ [31st aa, AAG (lys) to TAG] and 200 pg *faf1*, *grb14*, *sgcd*, *ap2b1*, or *galnt2* mRNA was injected into the yolk of 1-cell-stage embryos, respectively.

### DNA isolation and Southern blot analysis

DNA was extracted from zebrafish embryos using a DNA extraction kit according to the manufacturer’s protocol (AidLab DN08). The isolated genomic DNA was digested with EcoRV. The E*gfp* (226-646) DNA fragment was labeled with DIG as a probe for the southern blot assay. The southern blot assay was performed as described previously^64^.

### Linker-mediated PCR for identification of pIDM insertion sites

Linker annealing (AluI or BfaI linker + and AluI or BfaI linker-) was performed according to the protocol described in a previous study^64^. Linker-mediated PCR was performed as described previously^65^.

The Tol2 5′ S1 primer was used to sequence the PCR products. The sequence of the PCR product was blasted with the database GRCz10. The sequence of genomic DNA with a high identity (>95%) to the PCR product was considered to be the insertion site. If the similarity between the PCR product and genomic sequences was less than 95%, the insertion site was considered as non-identified (N.D).

The details of the procedure are provided in Detailed protocol of Supplementary Materials.

### Generation of nid2a−/− mutant with CRISPR/Cas9

The zebrafish Cas9 expression plasmid pGH-T7-zCas9^66^ was kindly provided by Prof. Zhang Bo at College of Life Sciences, Peking University. The sequence of *nid2a* target site was: 5′-GGAAAAGGATCCATCTACTAC-3′ at exon2 of *nid2a*. The genomic region flanking gRNA target site was amplified with a pair of primers: *nid2a Cas9 ID* for and *nid2a Cas9 ID rev*. The amplified DNA fragment was digested with a restriction enzyme Msp1 and subjected to Sanger sequencing.

### Injection of dsRNAs

Gene specific forward and reverse primers either fused with or without a T7 promoter site (ATAATACGACTCACTATA) at the 5′ end were designed to amplify two DNA fragments from the region of each of six genes: E*gfp1-720*, *nid2a* 27-630, *faf1* 1-624, *grb14X*3 132-789, *ap2b1* 170-792 and *sgcd*1-621 (Supplementary Table S1). The two PCR fragments were used as the templates to respectively synthesize sense and antisense RNAs for each gene. For making dsRNA, equal amounts of newly synthesized sense and antisense RNAs were mixed with addition of the annealing buffer as described previously^32^.

One nl of 10 ng/ul of each dsRNA was injected into the cytoplasm of one-cell stage embryo.

### Phenotypical screens for cardiovascular mutants

F_2_ embryos (mixed with ubi-pIDM transgenic embryos and wild-type siblings) were subjected to Dox induction (1 μg/ml) from 6-12 hpf, and cardiovascular and/or circulation defects were scored at 24, 48, or 72 hpf. F_3_ embryos were further confirmed to show identical phenotypes to F_2_ embryos. Live images of mutants and wild-type siblings were taken under a fluorescence microscope (DM5000B; Leica, Germany).

### Tail-PCR for cloning responsible genes

To identify the insertion sites, we extracted genomic DNA from F_2_ embryos having evident cardiovascular and/or circulation defects as described (Tiangen). We used TAIL-PCR and Sanger sequencing to clone the flanking genomic sequences of the transposon insertion sites as reported previously^38^. Specific primers designed at the upstream (GL) and downstream (GR) of every transposon insertion site and vector primers designed at both of the vector ends (RP3, LP3) were used to perform genotype-phenotype correlation analyses. First, we chose about 16 F_2_ embryos with cardiovascular and/or circulation defects to clone every insertion and affected genes. For example, if the transgenic line has three insertions, GL1, GL2, GL3 and GR1, GR2, GR3 primers designed at the zebrafish genome. Every embryo was amplified by PCR with GL1-LP3, GL2-LP3, GL3-LP3, GR1-LP3, GR2-LP3, and GR3-LP3, leading to cloning of the responsible insertions. Tail fins of F_2_ adults were genotyped with PCR to confirm their genotypes and insertions. We then crossed F_2_ transgenic adults with wild-type zebrafish, and identified vector insertions and phenotypes from F_3_ embryos. Finally, each genotype of the transgenic F_3_ embryos were validated by PCR with GL1-LP3, GL2-LP3, GL3-LP3 and GR1-LP3, GR2-LP3, GR3-LP3. The primers used for TAIL-PCR and genotyping are shown in Supplementary Table 1.

## Electronic supplementary material

Supplementary figures and information
