# Supplementary material for: A novel inducible mutagenesis screen enables to isolate and clone both embryonic and adult zebrafish mutants
Source: Sci Rep. 2017 Sep 4;7:10381. doi: 10.1038/s41598-017-10968-w (PMC5583359; doi:10.1038/s41598-017-10968-w)
Supplement: Supplementary file 1 — Supplementary figures and information [file 41598_2017_10968_MOESM1_ESM.pdf]

A novel inducible mutagenesis screen enables to isolate and clone both embryonic and adult zebrafish mutants

Zhipeng Ma[1a], Peipei Zhu[1a], Meijun Pang[3a], Liwei Guo[1], Nannan Chang[3], Jiyuan Zheng[3], Xiaojun Zhu[3], Ce Gao[2], Honghui Huang[5], Zongbin Cui[4], Jing-Wei Xiong[3*] Jinrong Peng[2*], Jun Chen[1*]

[1]Key laboratory for Molecular Animal Nutrition, Ministry of Education, Innovation Center for Signaling Network, College of Life Sciences, [2]College of Animal Sciences, Zhejiang University, 866 Yu Hang Tang Road, Hangzhou, China 310058; [3]Institute of Molecular Medicine, Beijing Key Laboratory of Cardiometabolic Molecular Medicine, and State Key Laboratory of Natural and Biomimetic Drugs, Peking University, Beijing, China 100871; [4]Key Laboratory of Aquatic Biodiversity and Conservation, Institute of Hydrobiology, Chinese Academy of Sciences, 8 Dong Hu Nan Road, Wuhan, Hubei, P. R. China 430072; [5]Key Laboratory of Freshwater Fish Reproduction and Development, Ministry of Education, State Key Laboratory Breeding Base of Eco-Environments and Bio-Resources of the Three Gorges Reservoir Region, School of Life Sciences, Southwest University, 2 Tiansheng Road, Beibei, Chongqing，China 400715.

[a]These authors contributed equally to this work.

Author Information: The authors declare no competing financial interest.

Correspondence and requests for materials should be addressed to J.C (chenjun2009@zju.edu.cn) or J.R.P (pengjr@zju.edu.cn) or J.W.X (jingwei_xiong@pku.edu.cn).

Supplementary information

Supplementary Figures

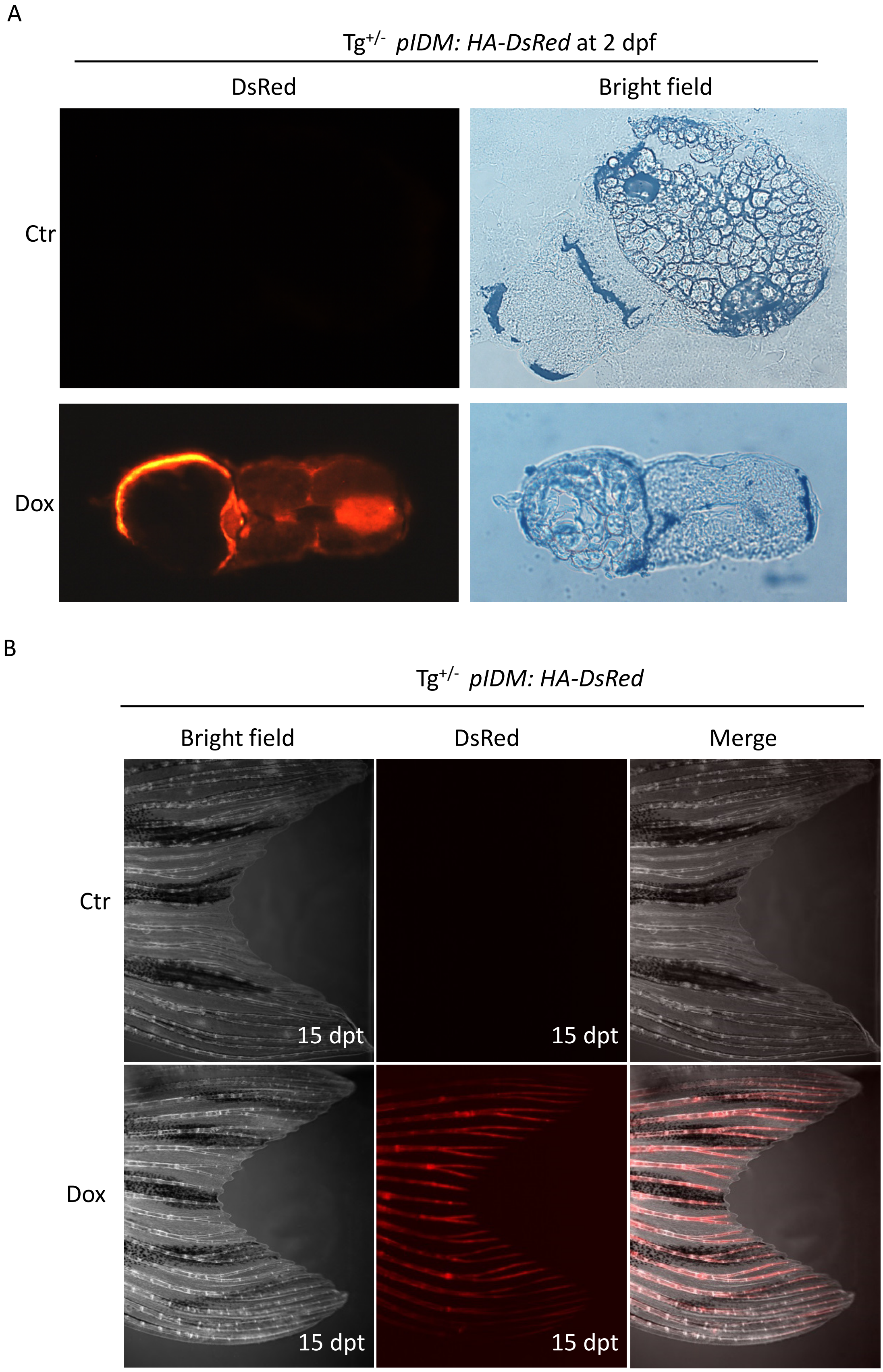

Supplementary figure 1

A

The first generation of pIDM

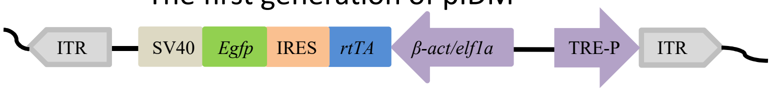

B

	Total No. of F0	No. of F0 with fluoresent progenies	Frequency of F0 with fluorescent progenies
pIDM without insulators	331	84	25.4%
pIDM with insulators	700	300	43%

C

pIDM line without insulators   pIDM line with insulators

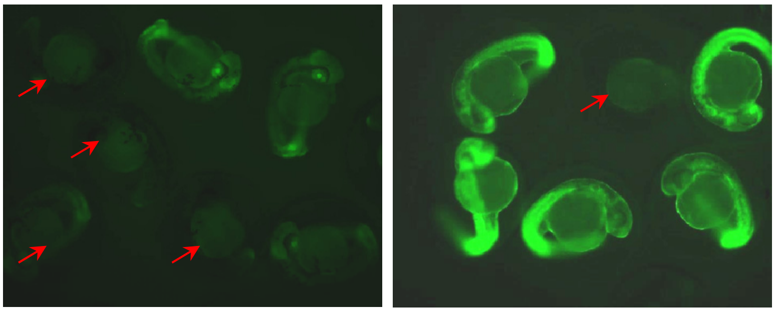

D

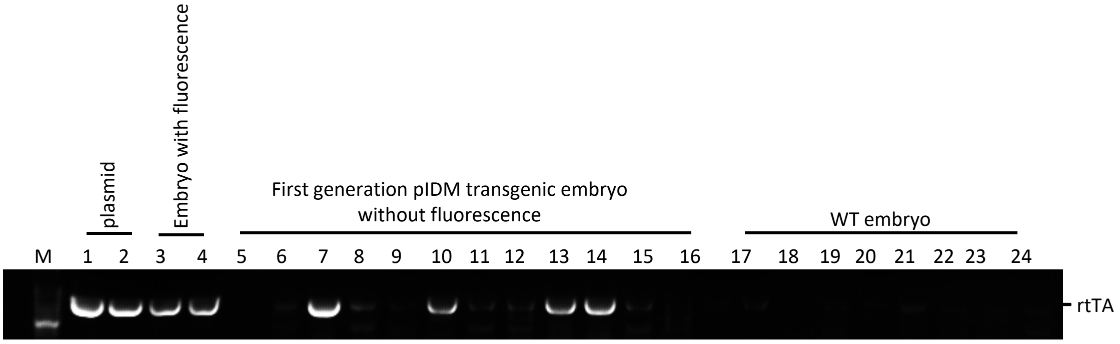

E

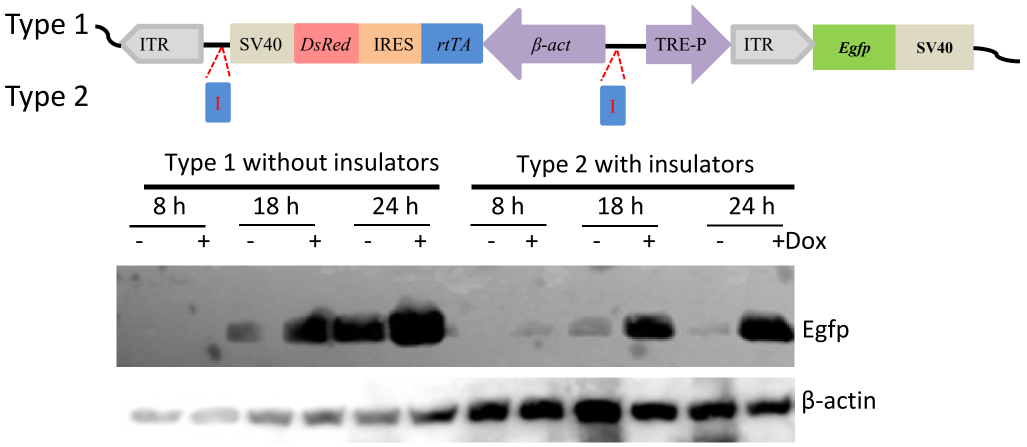

Supplementary figure 2

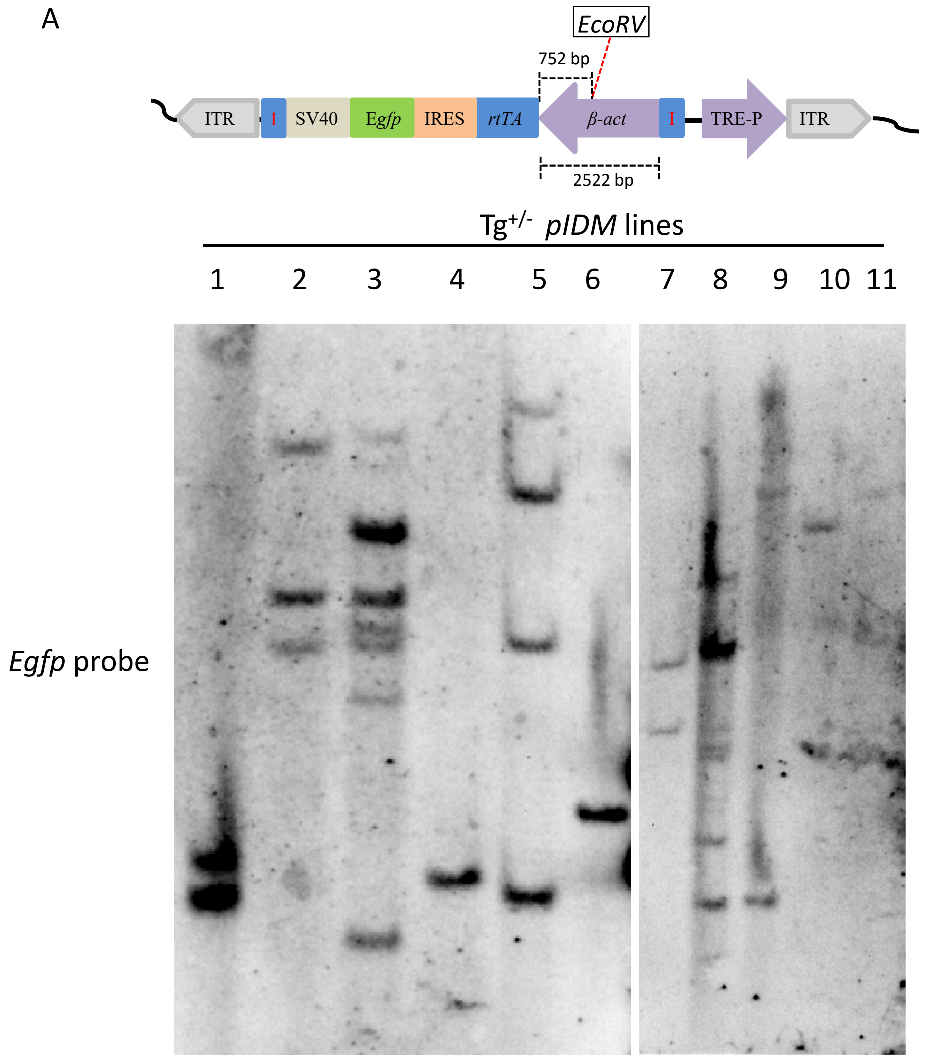

Supplementary figure 3

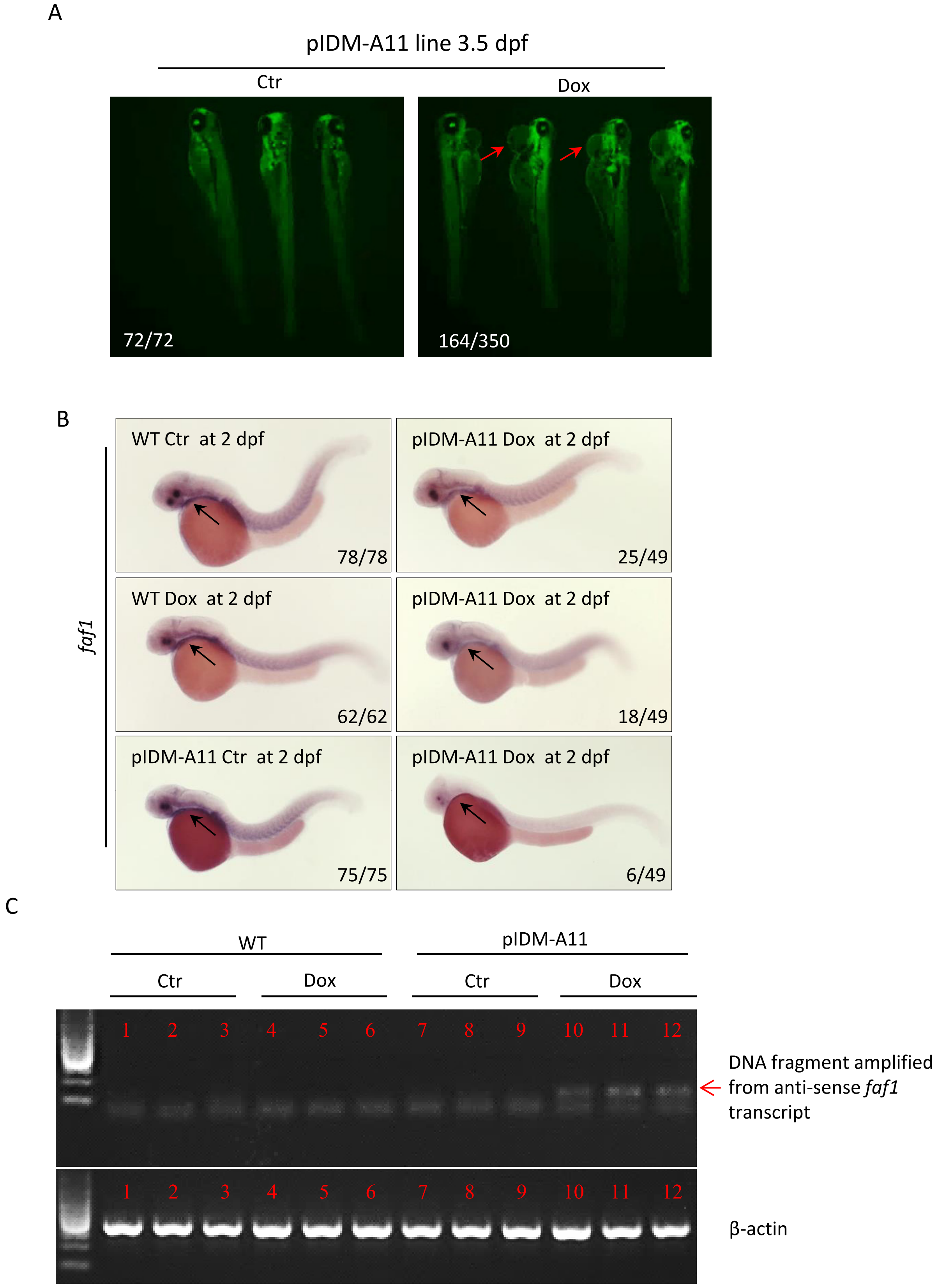

Supplementary figure 4

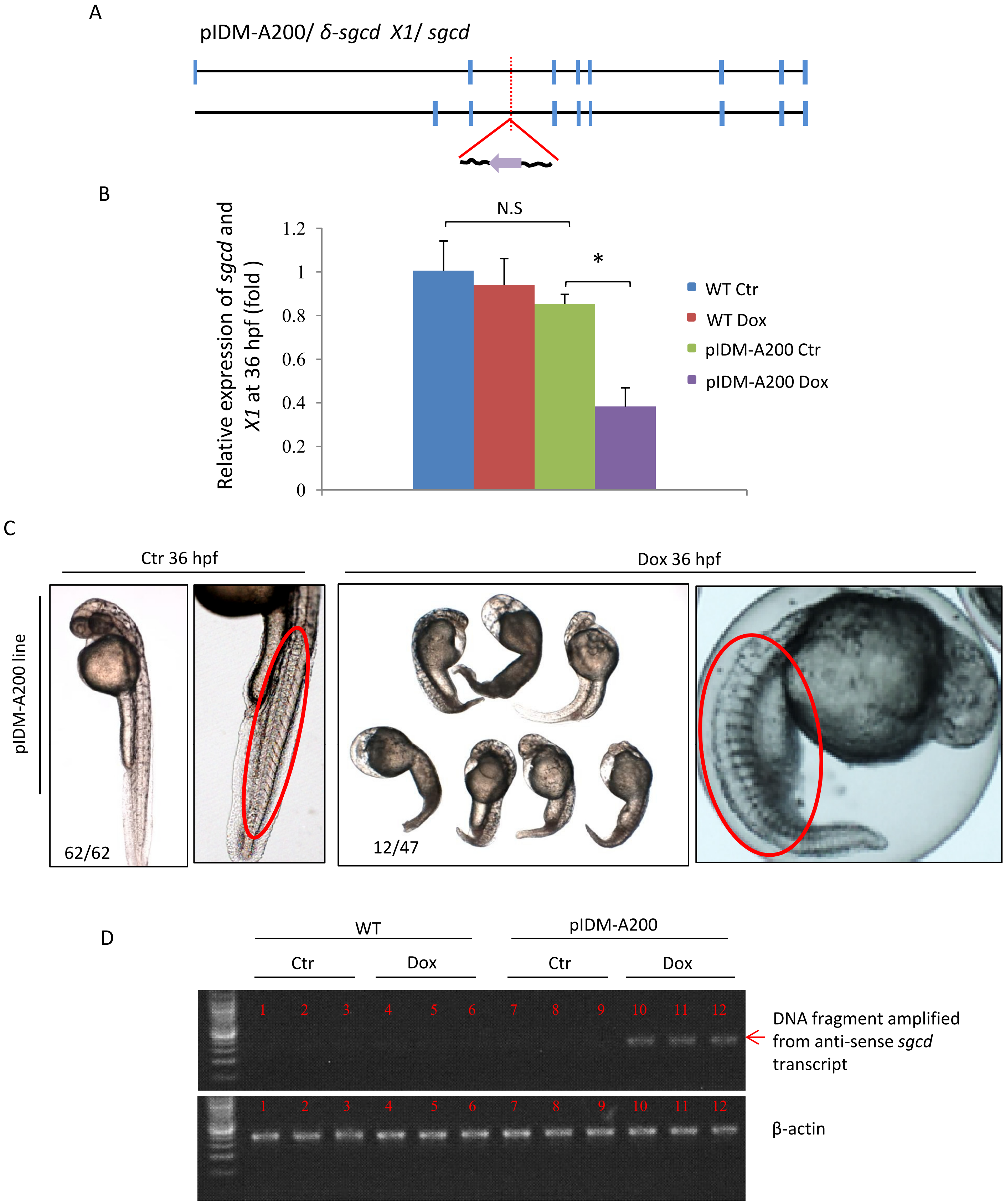

Supplementary figure 5

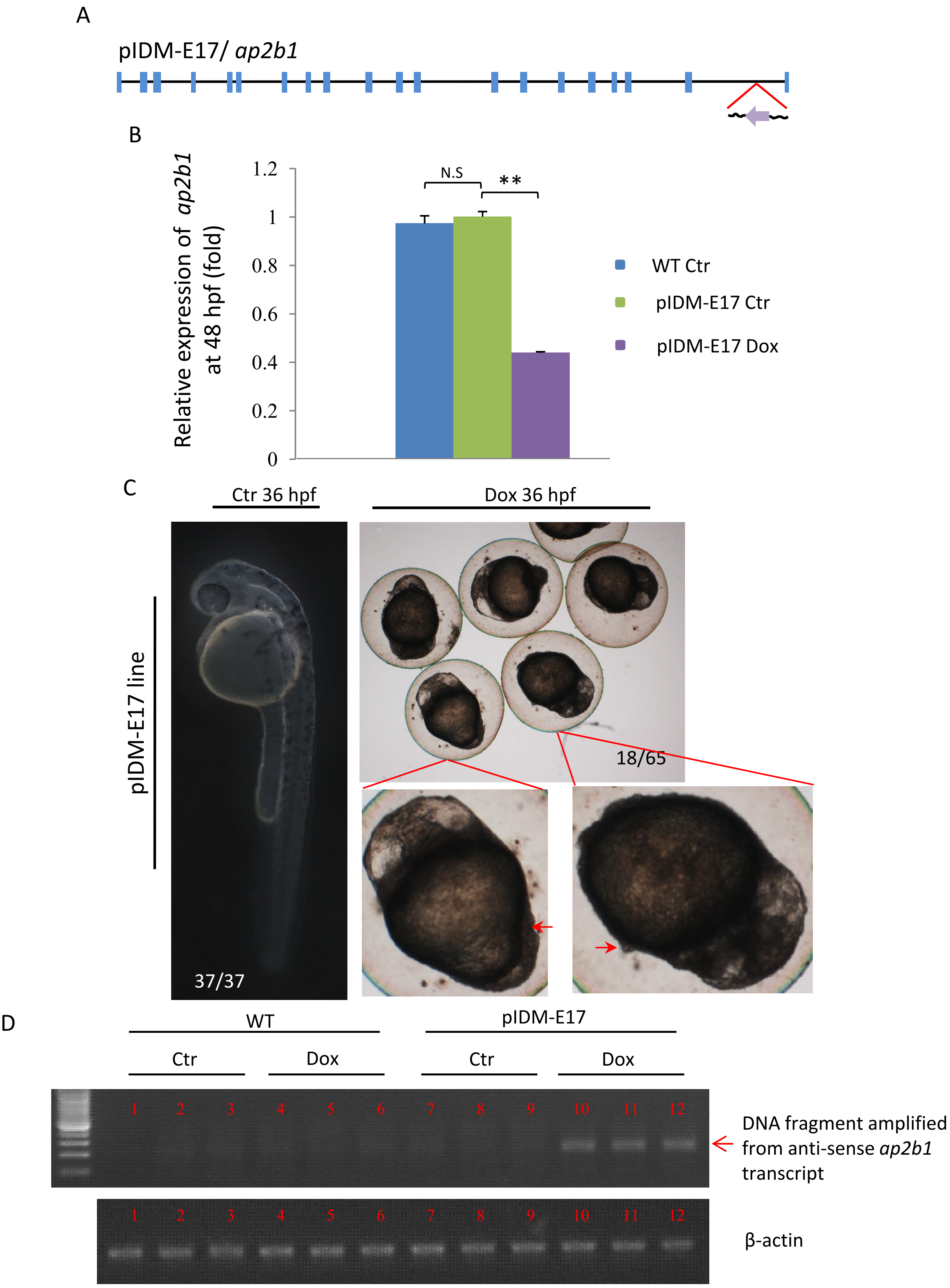

Supplementary figure 6

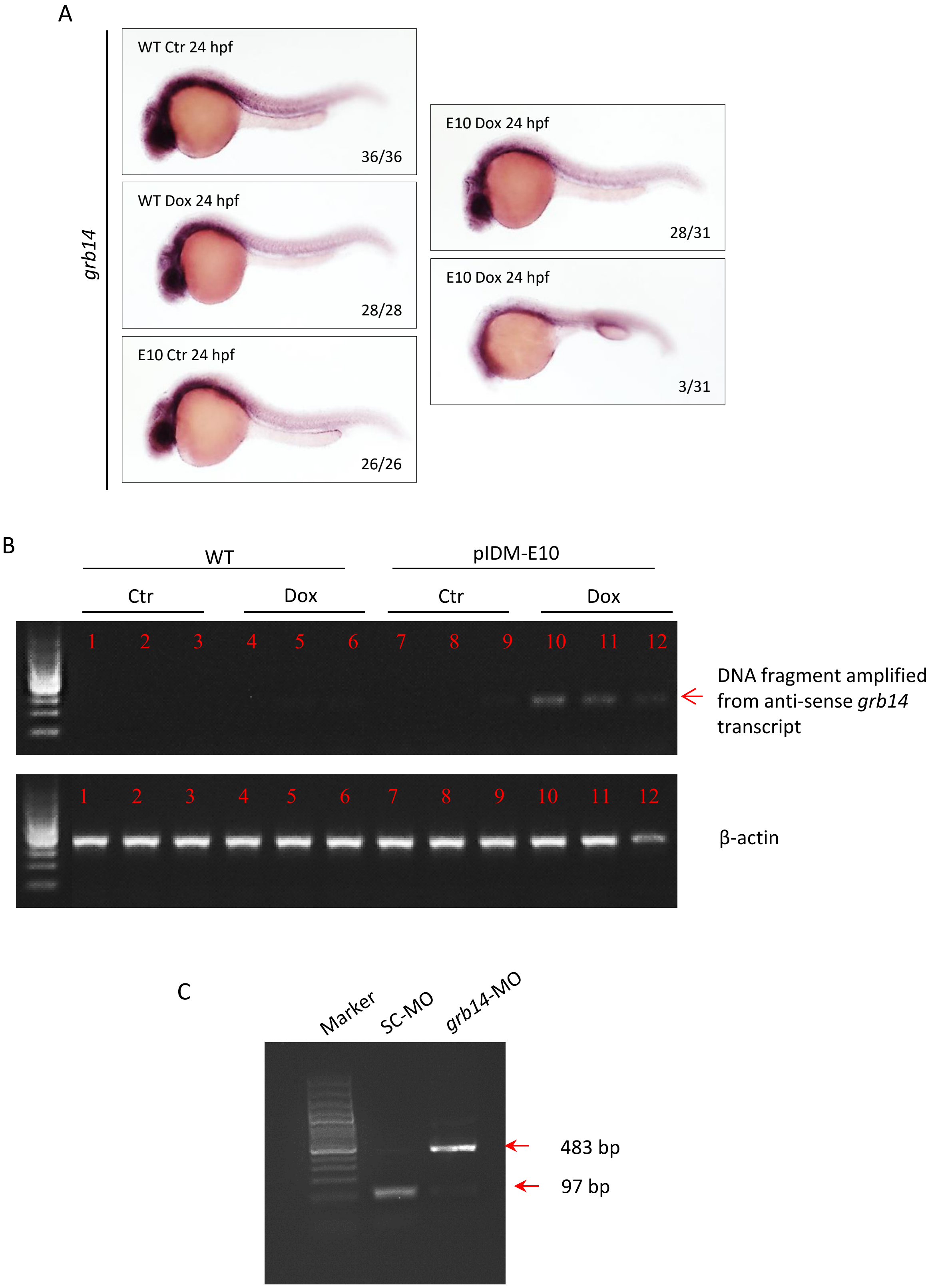

Supplementary figure 7

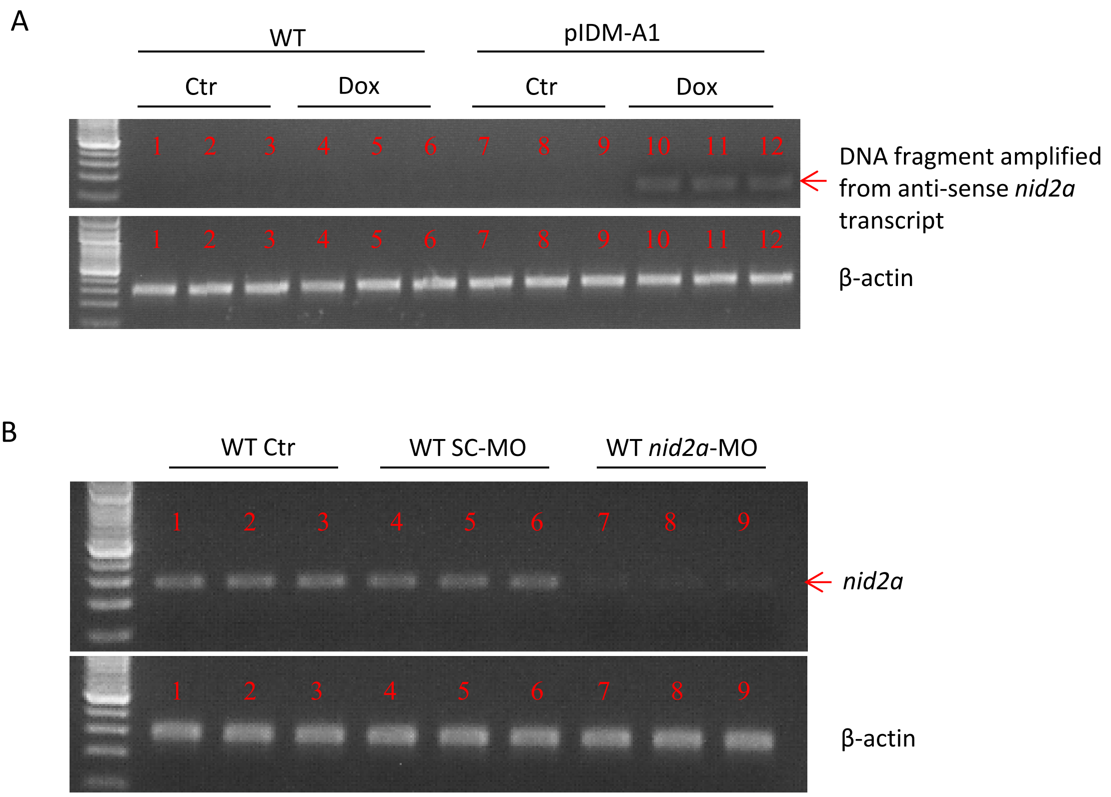

Supplementary figure 8

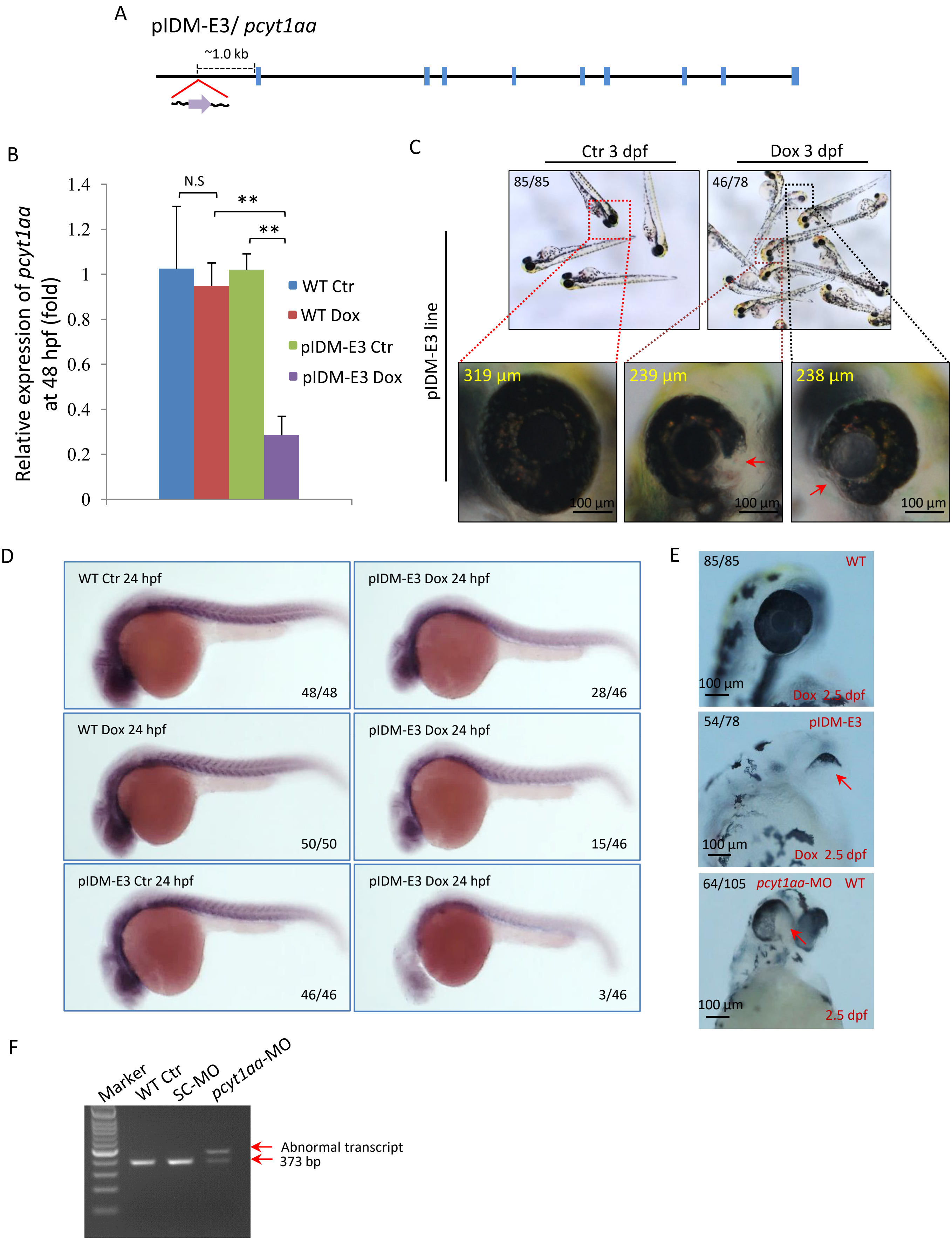

Supplementary figure 9

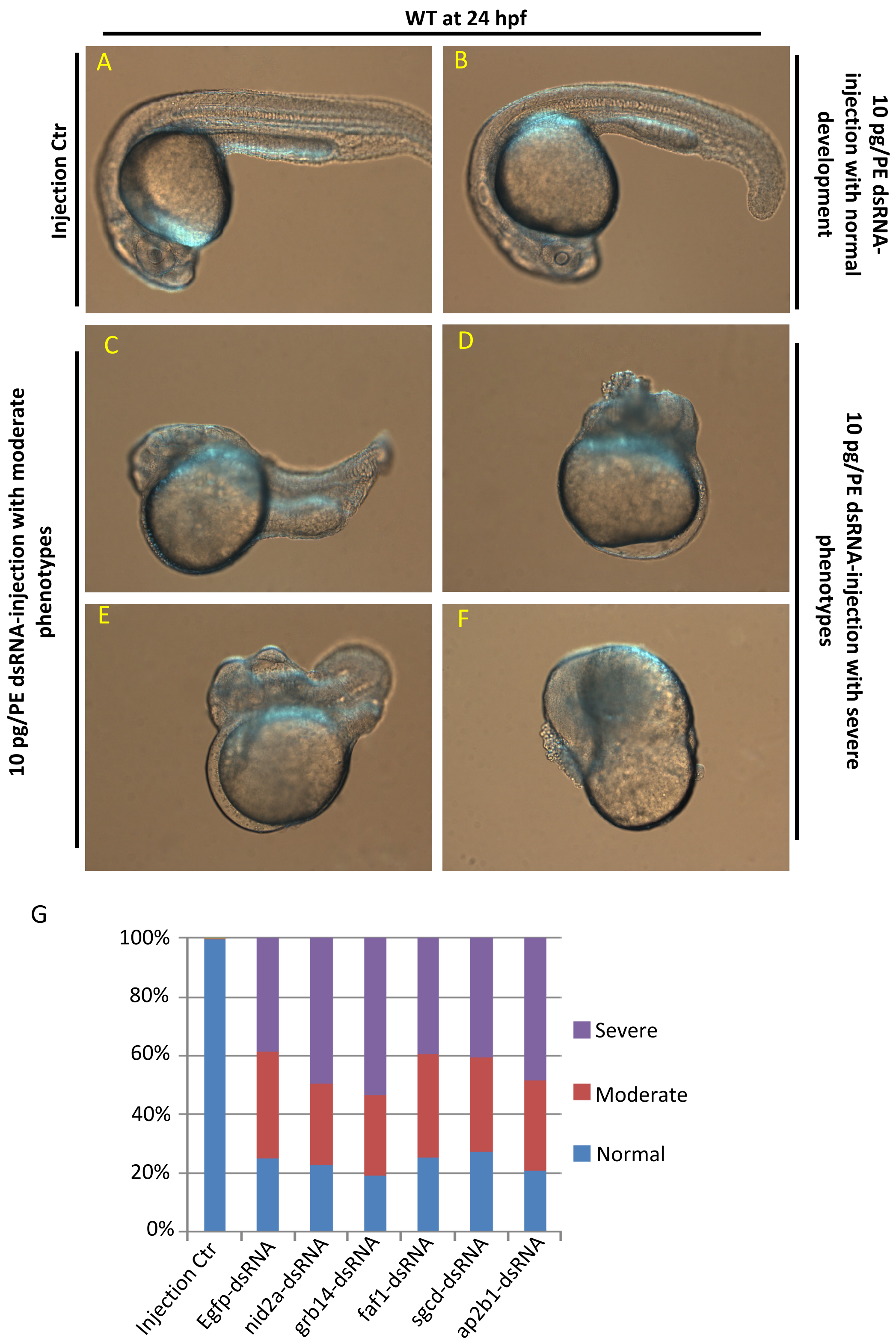

Supplementary figure 10

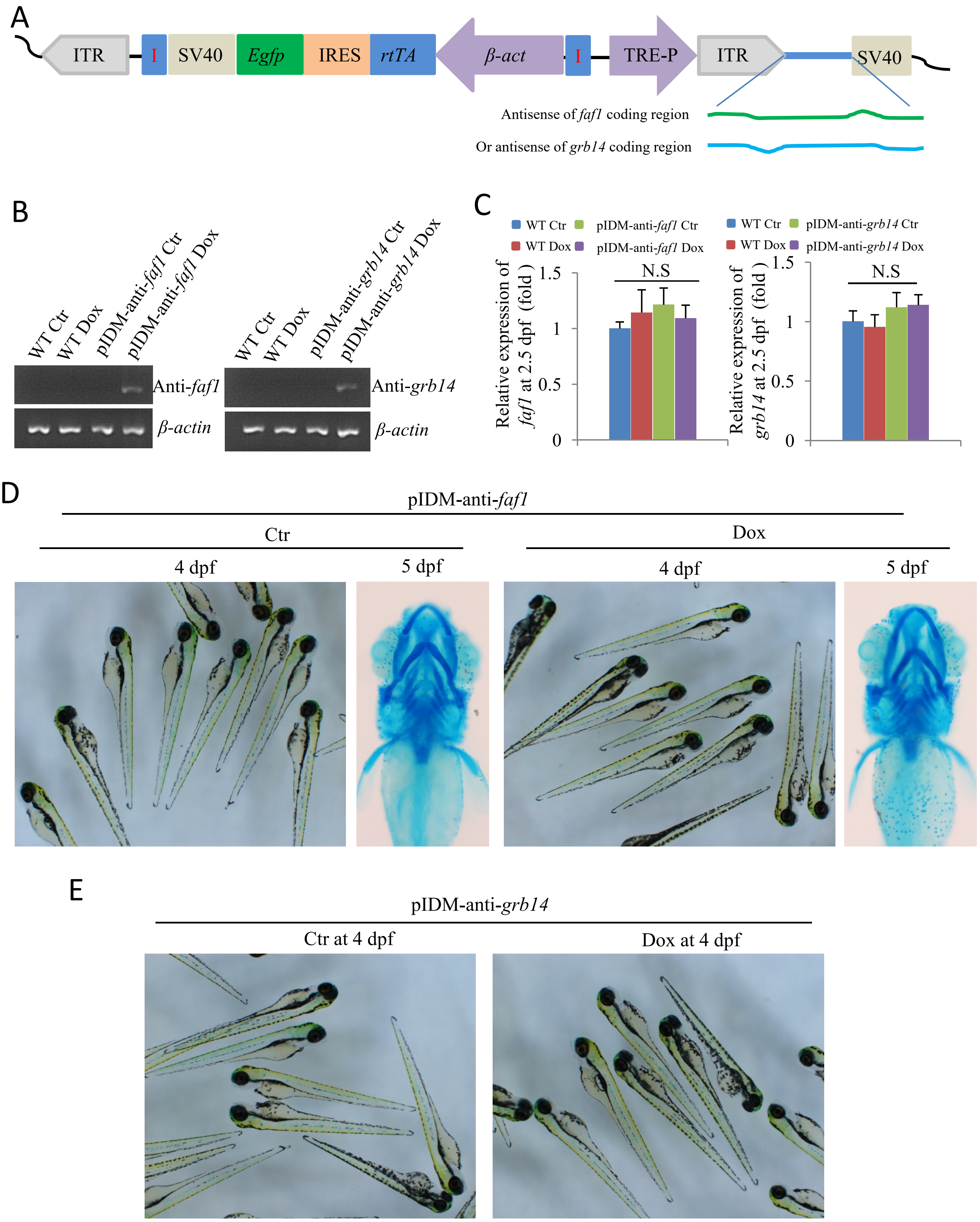

Supplementary figure 11

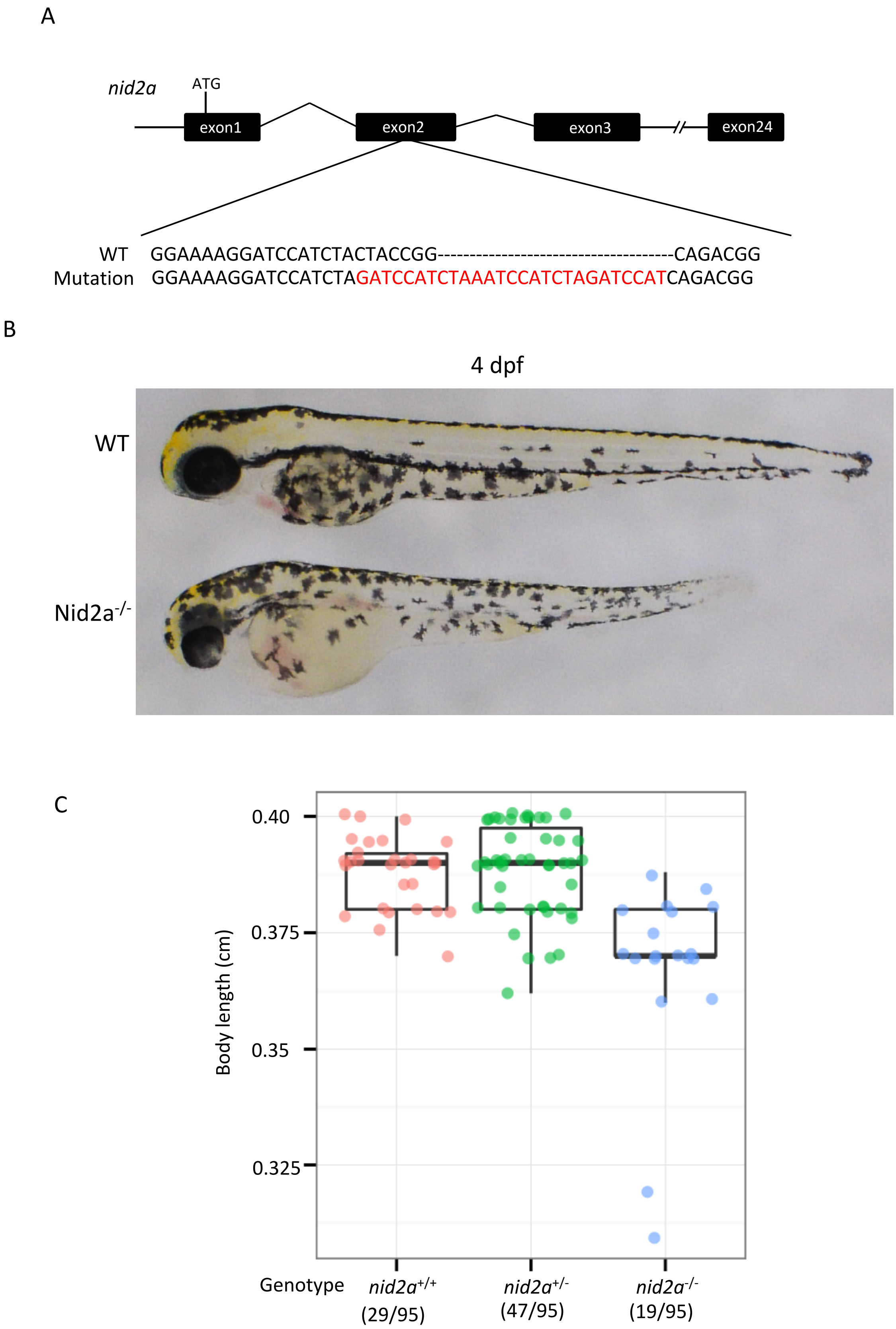

Supplementary figure 12

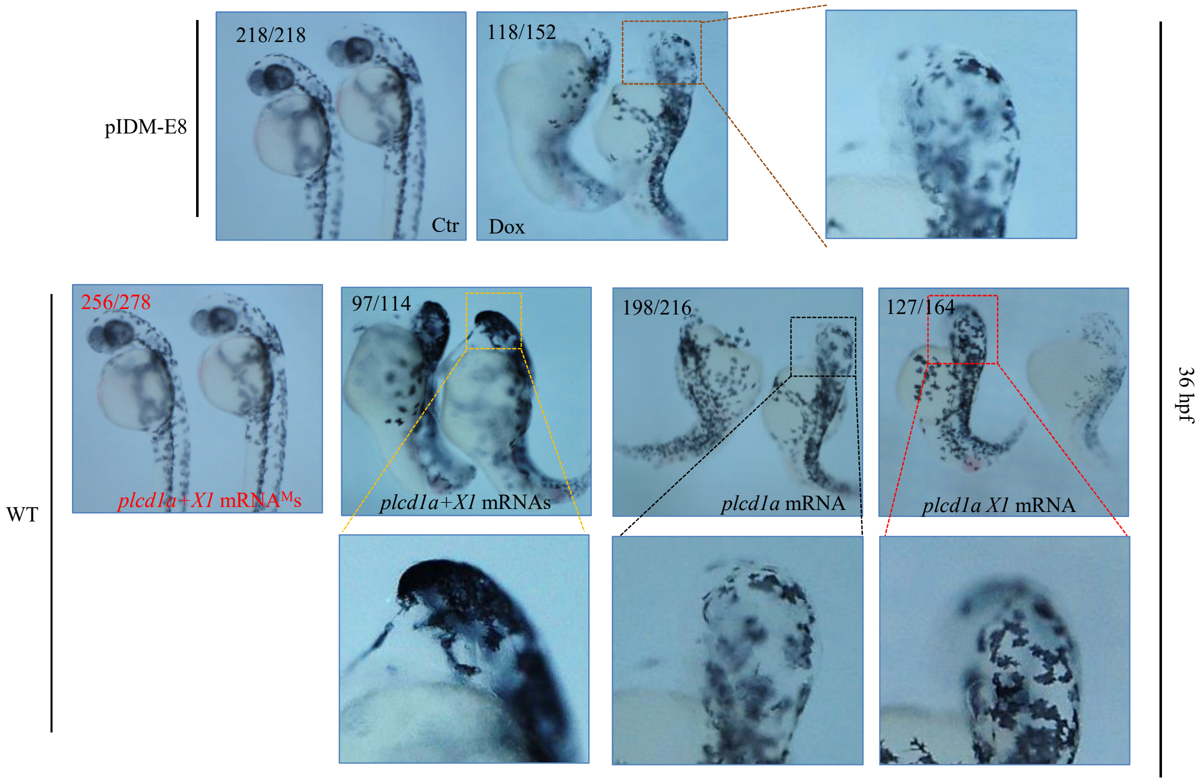

Supplementary figure 13

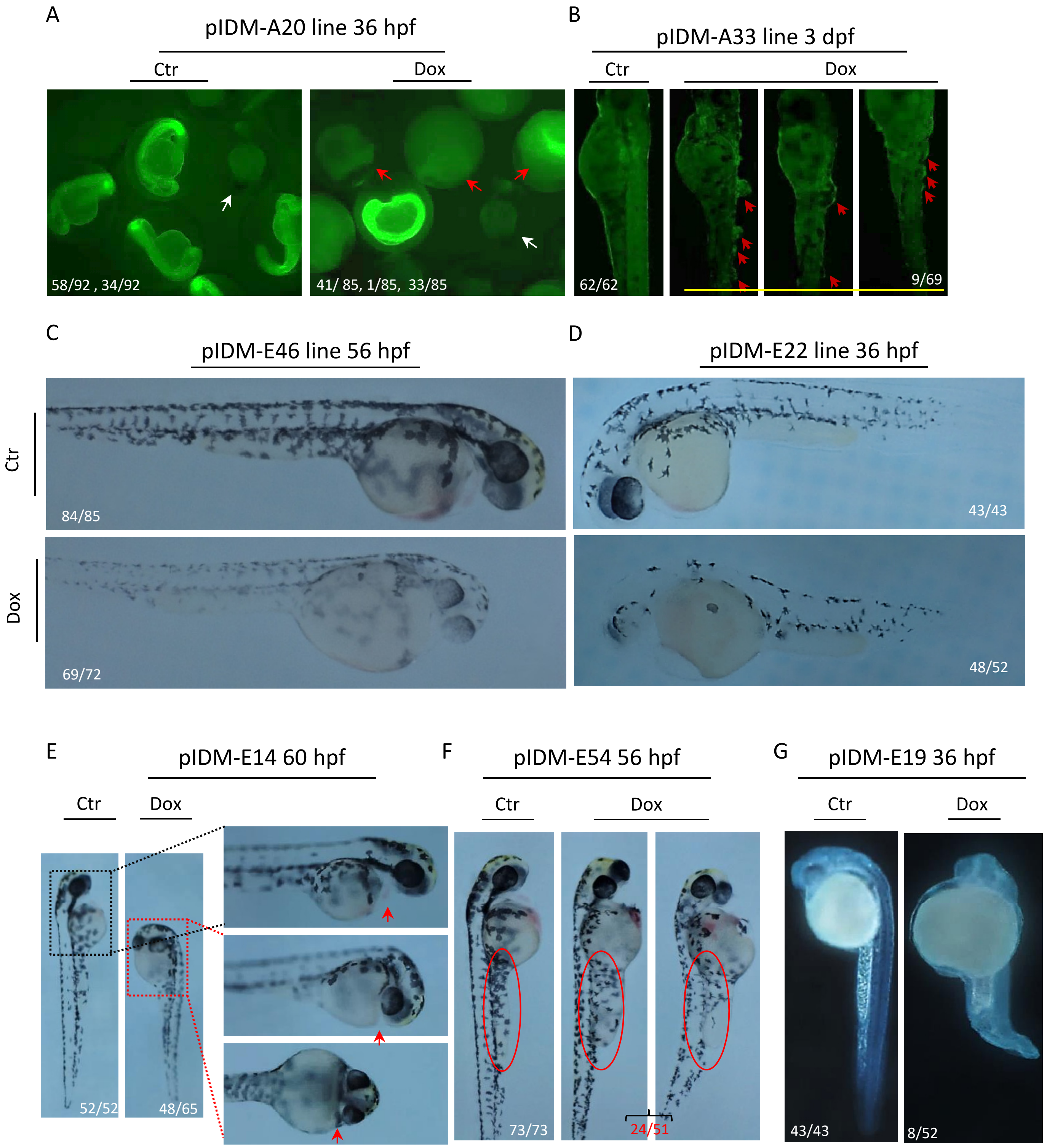

Supplementary figure 14-1

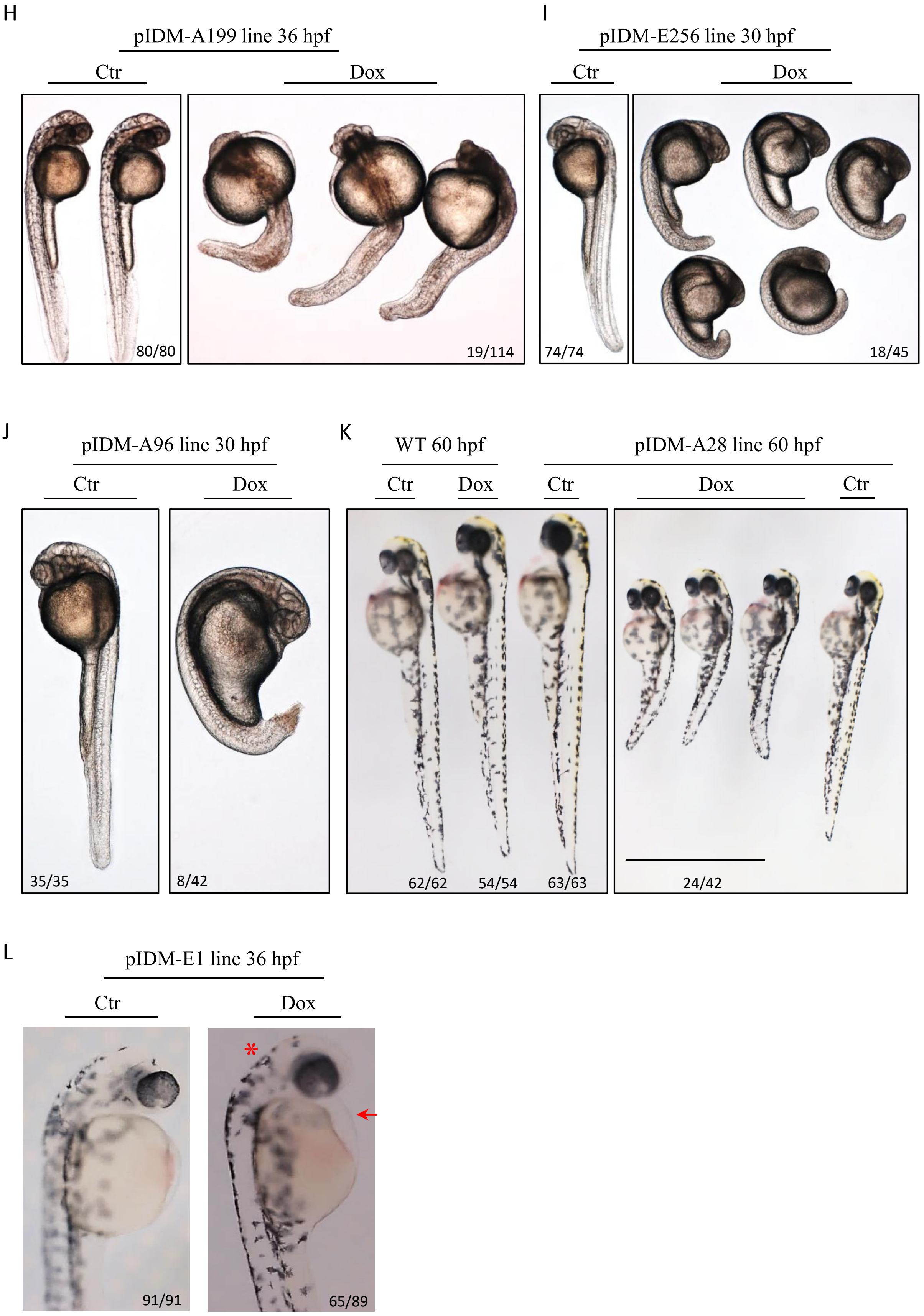

Supplementary figure 14-2

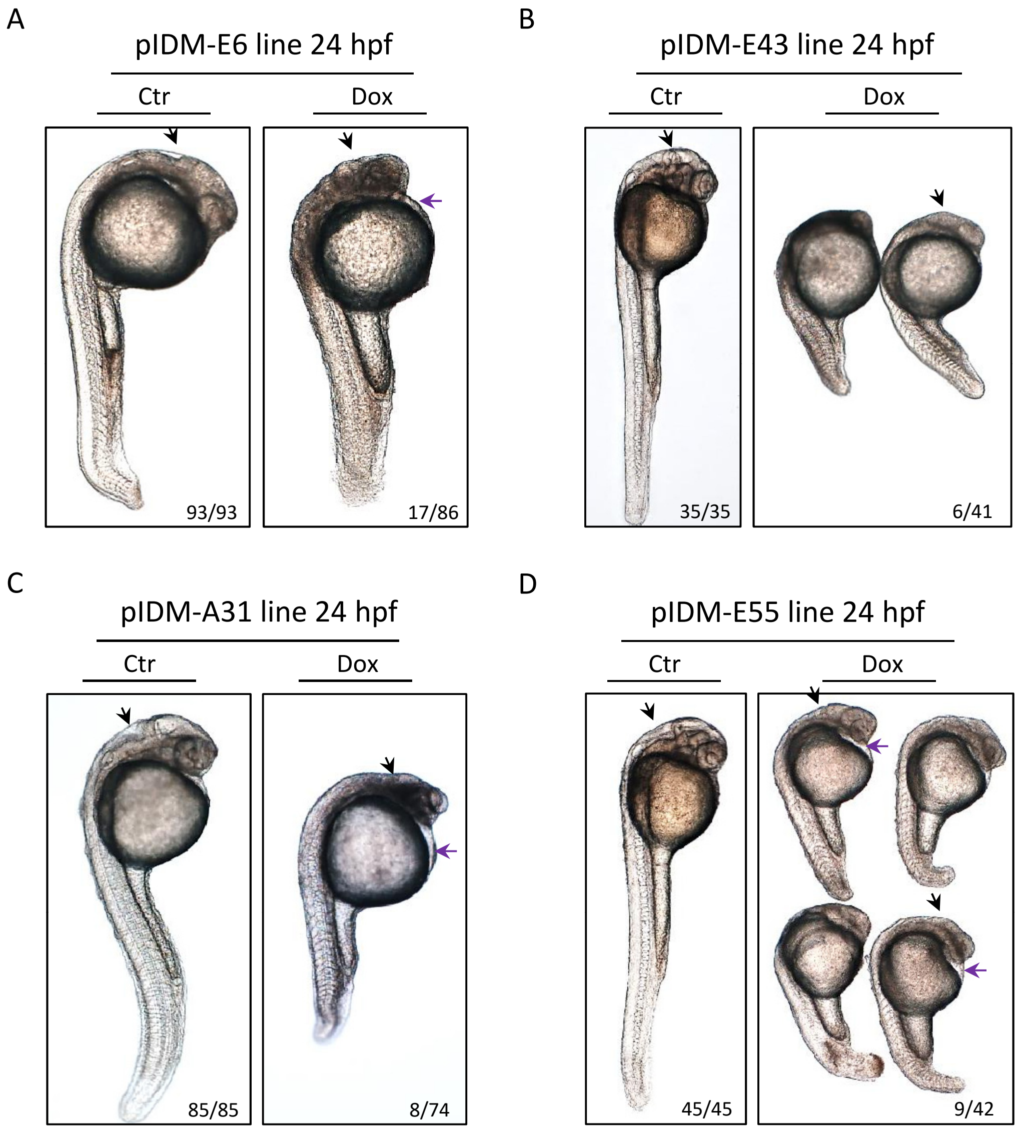

Supplementary figure 15

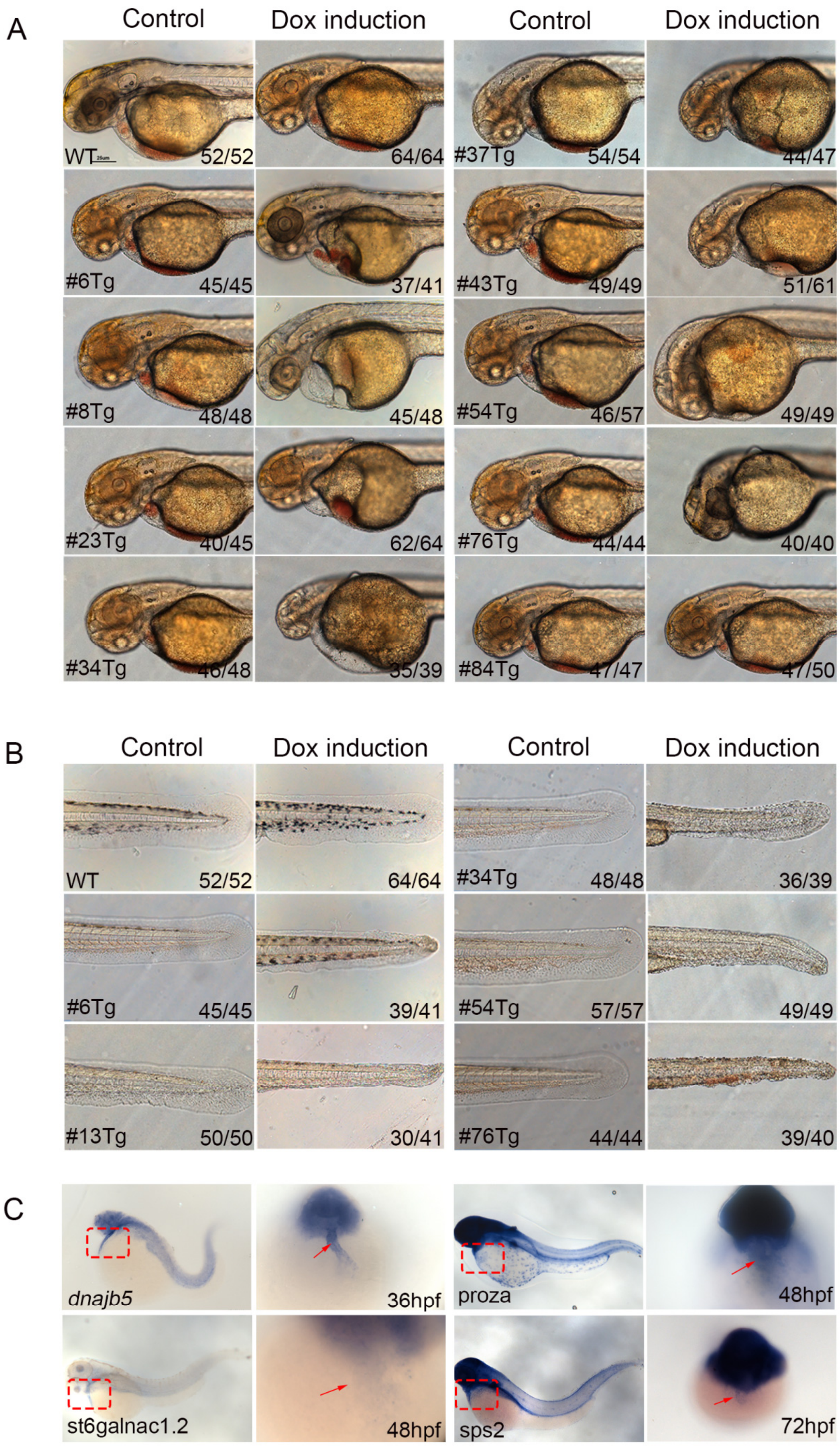

Supplementary figure 16

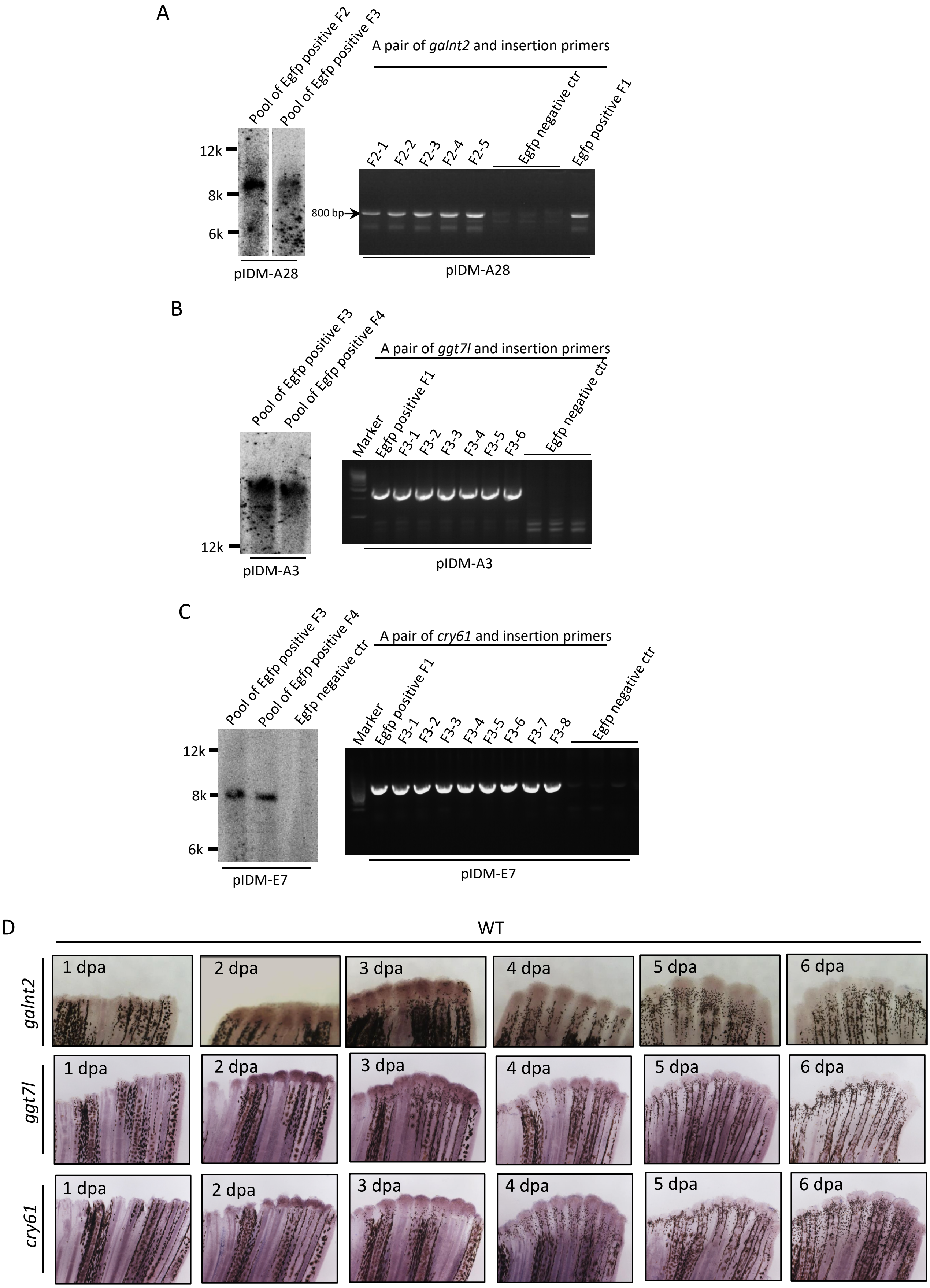

Supplementary figure 17

Supplemental Figure Legends

Supplementary Figure 1 The expression of HA-DsRed was induced by Dox treatment in the transgenic embryonic and adult fish harboring the modified *pIDM:HA-DsRed* transgene. (A) Transgenic zebrafish were generated as described in Figure 1B. Transgenic embryos were treated with Dox at 12 hpf. The treated and untreated embryos at 2 dpf were fixed with 4% PFA for 1 hour and then subjected to cryo-sections. The photo was taken with a fluorescent microscope. (B) Three-month-old transgenic fish were treated with Dox. The picture was taken under a confocal microscope at 15 dpt. $Tg^{+/-}$, heterozygous transgenic fish; Ctr, untreated control.

Supplementary Figure 2 Improvement of the pIDM system by including the combination of a pair of insulators. (A) Diagram of the first generation pIDM vector. The abbreviations for each item in the first generation pIDM construct are the same as those in the improved pIDM construct (Figure 1A), except where it lacks the pair of insulators. (B) Frequencies of F_0 founder fish with visible fluorescent F1 progenies. F_0 transgenic founder fish were generated with either the first generation or improved pIDM constructs. F_1 population containing visible green fluorescent embryos was considered to be positive transgenic lines. (C) The line on the left presented most transgenic lines generated with the first generation pIDM and the line on the right presented most transgenic lines generated with the improved pIDM. The picture was taken at 36 hpf. Red arrow, embryo without visible fluorescence. (D) Identification of transgene by PCR using *rtTA*-transgenic-fish-ID-for and *rtTA*-transgenic-fish-ID-rev primer pairs in F1 transgenic embryos generated with the first generation pIDM. Genomic DNA was extracted from WT embryos and F1 embryos either presence or absence of fluorescence. The plasmid DNA of pIDM (plasmid) was used as a positive control. (E) The inclusion of the insulator in pIDM sharply decreased the leakage of the Tet-on promoter. Upper panel: Diagram showing two types of vectors under analysis, either without or with a pair of insulators. In this construct, we used DsRed as the reporter gene and E*gfp* as the inducible

gene. Lower panels: Western blot analysis of EGFP. The plasmid was injected into WT embryos at the one-cell stage. The injected embryos were treated with Dox at 12 hpf. Total protein was extracted at 8, 18 or 24 hpt. An EGFP antibody was used to detect EGFP protein. β-actin was used as the protein loading control.

Supplementary Figure 3 Analysis of transgenic lines harboring the improved pIDM. (B) Southern blot analysis of insertion copy numbers in transgenic lines. Genomic DNA was extracted from pooled F_1 embryos of each line (a total of eleven lines examined, lane 1-11) and digested with the restriction enzyme EcoRV, which is located at the *β-actin* promoter. Number in the diagram, the position of EcoRV at the *β-actin* promoter. A digoxin (DIG) labeled *Egfp* fragment was used as the probe for the southern blot assay.

Supplementary Figure 4 Supplementary information for line pIDM-A11. (A) The F_1 pIDM-A11 embryos were treated with Dox at 12 hpf. The picture was taken at 3.5 dpf. Red arrow: pericardial edema. Number, type of represented embryos /total embryos in F_1 population. (B) Reduction of the *faf1* expression in the pIDM-A11 embryos treated with Dox. The treated embryos were sampled at 2 dpf and subjected to whole mount *in situ* hybridization (WISH). Anti-sense RNA of *faf1* was used to perform WISH. Black arrow, pharyngeal arch. Number, type of represented embryos/total embryos in F_2 population. (C) Induction of anti-sense transcript of *faf1* in the pIDM-A11 embryos treated with Dox. Total RNA was sampled at 2 dpf. The anti-sense *faf1* transcript was analyzed with quantitative reverse transcription PCR (qRT-PCR). The qRT-PCR in each treatment was repeated three times. *β-actin* was used as the qRT-PCR positive control. Red arrow, DNA fragment was amplified from anti-sense *faf1* transcript.

Supplementary Figure 5 Supplementary information for line pIDM-A200. (A) Diagram showing the position and orientation of pIDM in the *δ-sgcd* genomic DNA (including two isoforms, *δ-sgcd* and *X1*). (B) The relative expression levels of total *sgcd* and *X1* transcripts

were analyzed with specific primers at 48 hpf. *β-actin* was used to normalize the total RNA. (C) Pictures of pIDM-A200 embryos treated or untreated with Dox at 36 hpf. The enlarged pictures showed the abnormal muscular development phenotype (red oval). Number, type of represented embryos/total embryos in F_2 population. (D) The induction of the anti-sense transcript of *sgcd* in the pIDM-A200 embryos treated with Dox was analyzed with qRT-PCR using primer pairs *sgcd*-intron2-F and *sgcd*-intron2-R.

For all analysis on the relative expression of the target genes the statistically significant differences between samples were assessed using independent-sample *T*-tests (*$P<0.05$, **$P<0.01$, ***$P<0.001$).

Supplementary Figure 6 Supplementary information for line pIDM-E17. (A) Diagram showing the position and orientation of pIDM in the *ap2b1* genomic DNA. (B) The relative expression level of the *ap2b1* transcripts was analyzed with specific primers *ap2b1*-F and *ap2b1*-R at 48 hpf. *β-actin* was used to normalize the total RNA. (C) Pictures of pIDM-E17 embryos treated or untreated with Dox at 36 hpf. Red arrow, extruded-yolk. Number, type of represented embryos/total embryos in F_2 population. (D) The induction of the anti-sense transcript of *ap2b1* in the pIDM-E17 embryos treated with Dox was analyzed with qRT-PCR using primer pairs *ap2b1*-intron21-F and *ap2b1*-intron21-R.

Supplementary Figure 7 Supplementary information for line pIDM-E10. (A) Reduction of the *grb14* expression in the pIDM-E10 embryos treated with Dox. The treated embryos were sampled at 24 hpf and subjected to WISH. Anti-sense RNA of *grb14* was used to perform WISH. Number, type of represented embryos/total embryos in F_2 population. (B) Induction of the anti-sense transcript of *grb14* in the pIDM-E10 embryos treated with Dox was analyzed with qRT-PCR using the primer pairs *grb14*-intron2-F and *grb14*-intron2-R. (C) RT-PCR analysis showed that, due to abnormal splicing, the *grb14* morpholino (*grb14*-MO) had created an aberrant transcript with the extra 386 nt. WT embryos injected with the standard control morpholino (SC-MO) were used as the control.

Supplementary Figure 8 Supplementary information for line pIDM-A1. (A) The induction of the anti-sense transcript of *nidogen2a* in the pIDM-A1 embryos treated with Dox was analyzed with qRT-PCR using primer pairs *nidogen2*-intron9-F and *nidogen2*-intron9-R. (B) qRT-PCR analysis showed that *nid2a*-MO had decreased the expression of the normal transcript of *nidogen2a*, due to abnormal splicing. WT embryos and WT embryos injected with SC-MO were used as the control.

Supplementary Figure 9 Supplementary information for line pIDM-E3. (A) Diagram showing the position and orientation of pIDM in the *pcyt1aa* genomic DNA. (B) The relative expression levels of the *pcyt1aa* transcript were analyzed with specific primers *pcyt1aa*-F and *pcyt1aa*-R at 48 hpf. *β-actin* was used to normalize the total RNA. (C) Pictures of pIDM-E3 embryos treated or untreated with Dox at 3 dpf. The diameter of the eyes in the enlarged pictures is shown as a yellow number. Red arrow, less pigmentation in eyes. (D) Reduction of the *pcyt1aa* expression in the pIDM-E3 embryos treated with Dox. The treated embryos were sampled at 24 hpf and subjected to WISH. Anti-sense RNA of *pcy1aa* was used to perform WISH. (E) Pictures of WT and pIDM-E3 embryos with different treatments at 2.5 dpf as indicated. *pcyt1aa*-MO was designed to specifically block *pcyt1aa* transcript splicing and was injected into WT embryos at the one-cell stage. Red arrow, less pigmentation in eyes. (F) RT-PCR analysis showed that *pcyt1aa*-MO had created an aberrant transcript of *pcyt1aa*, due to abnormal splicing. WT embryos and WT embryos injected with SC-MO were used as the control.

Numbers in C-E, type of represented embryos/total embryos in F_2 population.

Supplementary Figure 10 Embryonic abnormalities caused by dsRNA injection. (A-F) WT embryos were injected with 10 pg/per embryo (pg/PE) *Egfp dsRNA* or *nid2a*, *grb14*, *faf1*, *sgcd*, *ap2b1* dsRNA at the one-cell stage. Embryos injected phenol-red buffer as the injection

control (A). Most of embryos injected with all of the 6 dsRNAs showed similar abnormal development. The similar abnormal development can be divided into three categories: Normal development with no obvious phenotypes (B), Moderate phenotypes (C, E) and Severe phenotypes (D, F). (G) Statistics of different types of embryos in each treatment with dsRNA injection. Approximately 300 injected embryos were counted in each treatment.

Supplementary Figure 11 Dox treated transgenic embryos from either pIDM-anti-*faf1* or pIDM-anti-*grb14* developed normally and did not display similar phenotypes as Dox treated mutant embryos from either pIDM-A11 or pIDM-E10 did. (A) Diagram of pIDM-anti-*faf1* and pIDM-anti-*grb14* vectors. The abbreviations for each item in the vectors are the same as those in the *pIDM:HA-DsRed* construct (Figure 1B), except where *HA-DsRed* was replaced with either the anti-sense of *faf1* or *grb14* coding region cDNA. (B) Induction of the anti-sense transcript of *faf1* or *grb14* in the pIDM-anti-*faf1* or pIDM-anti-*grb14* embryos treated with Dox was analyzed with qRT-PCR using the primer pairs anti-*faf1*-qRT-For and –Rev or anti-*grb14*-qRT-For and -Rev. (C) The relative expression level of the *faf1* or *grb14* transcripts was analyzed with specific primers *faf1*-F and *faf1*-R or *grb14-F* and *grb14-R* in different samples as indicated at 2.5 dpf . *β-actin* was used to normalize the total RNA. (D) The pIDM-anti-*faf1* embryos were treated with Dox at 12 hpf. The pictures were taken at 4 dpf (1st and 3rd panels). The embryos treated and untreated with Dox (Ctr) were sampled at 5 dpf and subsequently subjected to alcian-blue-staining for pharyngeal cartilage (2nd and 4th panels). (E) The pIDM-anti-*grb14* embryos were treated with Dox at 12 hpf. The pictures were taken at 4 dpf.

Supplementary Figure 12 *nid2a* knockout mutants display similar phenotypes to those in pIDM-A1 embryos upon Dox treatment. (A) Upper panel: Diagram showing the genome structure of *nid2a* and the gRNA target site. Bottom panel: Comparison of genomic DNA among WT and *nid2a*$^{-/-}$ mutant (with a 7 bp-deletion and a 27 bp-insertion in the 2nd exon). ATG: translation start codon. The mutation will lead to an early stop codon. (B) Picture of WT and *nid2a* homozygous mutant (*nid2a*$^{-/-}$) embryos at 4 dpf. (C) Statistics of body length (centimeter cm) of different genotype embryos in a F_2 population at 4 dpf as indicated. Numbers: embryos with the genotype versus total embryos in the F_2 population examined.

Supplementary Figure 13 Characterization of line pIDM-E8. Pictures of WT and mutant embryos with differing treatments at 36 hpf as indicated. For mRNA injection, WT embryos were injected with *plcd1a* mRNA, or *plcd1aX1* mRNA, or *plcd1a* together with *plcd1aX1* mRNAs (*plcd1a+X1* mRNAs), or both of the mutant mRNAs carrying an early stop codon (*plcd1a+X1* mRNA^{M}s) at the one cell stage. Enlarged pictures to show delayed eye's development. Numbers: embryos showing the displayed phenotype versus total embryos examined are provided in the corresponding pictures.

Supplementary Figure 14 Morphology of Dox treated pIDM mutants with multiple insertions. (A) Line pIDM-A20. Early embryonic lethality for Dox treated pIDM-A20 embryos at 36 hpf. Red arrow, dead embryos; white arrow, WT embryos. In untreated pIDM-A20 embryos, 58 embryos were EGFP positive and 34 embryos were EGFP negative. None of these embryos had died. In Dox treated pIDM-A20 embryos (total 85), 41 embryos were dead at 36 hpf, while among 34 living embryos, only one embryo (with clearly retarded

development) was EGFP positive with 33 embryos EGFP negative. (B) Line pIDM-A33. Epidermal blisters in Dox treated pIDM-A33 embryos at 3 dpf. Red arrow, lesions. (C) Line pIDM-E46. Less pigmentation in Dox treated pIDM-E46 embryos at 56 hpf. (D) Line pIDM-E22. Less pigmentation in Dox treated pIDM-E22 embryos at 36 hpf. (E) Line pIDM-E14. Short stature and no pericardium in Dox treated pIDM-E14 embryos at 60 hpf. Red arrow, pericardium. (F) Line pIDM-E54. Shorter and thicker yolk extension in Dox treated pIDM-E54 embryos at 56 hpf. Red ellipse, yolk extension. (G) Line pIDM-E19. Small head, curved body and unabsorbed yolk in Dox treated pIDM-E19 embryos at 36 hpf. (H) Line pIDM-A199. Curved body with severe cell death in Dox treated pIDM-A199 embryos at 36 hpf. (I) Line pIDM-E256. Arrested development in Dox treated pIDM-E256 embryos at 30 hpf. (J) Line pIDM-A96. Arrested development in Dox treated pIDM-A96 embryos at 30 hpf. (K) Line pIDM-A28. Short stature in Dox treated pIDM-A28 embryos at 60 hpf. (L) Line pIDM-E1. Pericardial edema and deformed head in Dox treated pIDM-E1 embryos at 36 hpf. Red arrow, pericardial edema; (*), deformed head.

Number in B-L, type of represented embryos/total embryos in F_2 population.

Details of insertion positions, orientation of pIDM and expression changes of tagged genes in each line are provided in Table 1.

Supplementary Figure 15 Non-specific phenotypes in Dox treated pIDM mutants with multiple insertions. No-midbrain-hindbrain boundaries, lack of normal brain ventricles, defects in eye development and pericardial edema were observed in Dox treated embryos of pIDM-E6 (A), pIDM-E43 (B), pIDM-A31 (C) and pIDM-E55 (D) lines. Black arrow, brain-boundaries; purple arrow, heart edema.

Number in A-D, type of represented embryos/total embryos in F_1 population.

Supplementary Figure 16. Morphological abnormalities of ubi-pIDM-transgenic embryos after Dox induction. (A-B) Nine mutant lines had Dox-induced cardiovascular defects (A) and five lines had abnormal blood circulation and tail defects (B). Either wild-type (WT; left) or

ubi-pIDM transgenic embryos (such as #6Tg; right) were treated with DMSO (control) or Dox (Dox-induction), and cardiovascular, tail and circulation phenotypes were documented at 48 hpf. The numbers on the lower right indicate phenotypical numbers out of the total embryos examined. The Scale bar: 25 m. (C) RNA *in situ* hybridization revealed that *Dnajb5*, *proza*, *St6galnac1.2,* and *sps2* were expressed in the heart on the left column (lateral view), and the ventral view of the heart area is enlarged on the right column. Arrows point to the heart. Staged embryos are noted for *dnajb5* at 36 hpf, for *proza* and *st6galnac1.2* at 48 hpf, and for *sps2* at 72 hpf.

Supplementary Figure 17. Segregation analysis of causative mutation from the multiple-inserted lines with Dox-dependent fin regeneration defects. (A) Segregation of $galnt2^{IM}$ from line pIDM-A28. To segregate the causative mutation, pIDM-A28 F_1 mutants with Dox-dependent fin regeneration defects were crossed with WT. The cross containing about 50% EGFP positive offspring was selected to produce F_2 population. Left panel: Southern blot analysis of insertion copy numbers in the pool of embryos as indicated. Pool of EGFP positive F_2: The F_2 EGFP positive embryos were from the cross between the selected F_1 fish and WT; Pool of EGFP positive F_3: The F_3 EGFP positive embryos were from the cross between one of F_2 adult fish and WT. Southern blot was performed as described in Supplementary Figure 3. Right panel: PCR analysis to identify the insertion site. A pair of *galnt2* intron 1 and pIDM specific primers were designed to amplify the insertion site. The DNA lysed from caudal fin of individual F_2 fish was used to do PCR analysis. The DNA from EGFP positive F_1 embryo pool was used as the positive control. The DNA from EGFP negative F_2 fish was used as the negative control. (B) Induction of the listed genes in WT zebrafish at different time points during fin regeneration as indicated. The WT caudal fin was sampled at different times after amputation and subjected to a WISH assay with the corresponding anti-sense RNA probes as indicated. (C) Segregation of $ggt7l^{IM}$ from line pIDM-A3. To segregate the causative mutation, pIDM-A3 F_2 mutants with Dox-dependent fin regeneration defects were crossed with WT. The cross containing about 50% EGFP positive

offspring were selected to produce F_3 population. Left panel: Southern blot analysis of insertion copy numbers in the pool of embryos as indicated. Pool of EGFP positive F_3: The F_3 EGFP positive embryos were from the cross between the selected F_2 fish and WT; Pool of EGFP positive F_4: The F_4 EGFP positive embryos were from the cross between one of F_3 adult fish and WT. Southern blot was performed as described in Supplementary Figure 3. Right panel: PCR analysis to identify the insertion site. A primer from *ggt7l* exon 5 was used with pIDM specific primer to amplify the insertion site. The DNA lysed from caudal fin of individual F_3 fish was used to do PCR analysis. The DNA from EGFP positive F_1 embryo pool was used as the positive control. The DNA from EGFP negative F_3 fish was used as the negative control. (D) Segregation of *cry61*^{IM} from line pIDM-E7. To segregate the causative mutation, pIDM-E7 F_2 mutants with Dox-dependent fin regeneration defects were crossed with WT. The cross containing about 50% EGFP positive offspring were selected to set up F_3 population. Left panel: Southern blot analysis of insertion copy numbers in the pool of embryos as indicated. Pool of EGFP positive F_3: The F_3 EGFP positive embryos were from the cross between the selected F_2 fish and WT; Pool of EGFP positive F_4: The F_4 EGFP positive embryos were from the cross between one of F_3 adult fish and WT. Southern blot was performed as described in Supplementary Figure 3. Right panel: PCR analysis to identify the insertion site. A primer from *cry61* intron 1 was used with pIDM specific primer to amplify the insertion site. The DNA lysed from caudal fin of individual F_3 fish was used to do PCR analysis. The DNA from EGFP positive F_1 embryo pool was used as the positive control. The DNA from EGFP negative F_3 fish was used as the negative control.

Detailed protocol

Plasmid construction

Construction of the first generation pIDM vector:

The first step: The *rtTA* DNA fragment was amplified with the primer pair *rtTA*-BamHI-F and -R from the pT2HB-PUHrT62 plasmid. The amplified *rtTA* DNA fragment was then digested with *BamHI* and cloned into a pIRES$_2$-EGFP plasmid (Clontech) to generate the prtTA-IRES2-EGFP construct. The prtTA-IRES2-EGFP construct was then used to amplify the *rtTA*-IRES-E*gfp*-*polyA* fragment with the primer pair *rtTA*-IRES-E*gfp*-*SV40*-NcoI-F and -R.

The second step: TRE3G promoter[1] was synthesized by the Biosune company and ligated into the pT2-*β-actin* plasmid to generate the pT2-*β-actin*-TRE3G vector. For the pT2-*elf1a*-TRE3G vector, an *elf1a* element was amplified from the T2KXIG plasmid with *elf1a*-HindIII-F and *elf1a*-NcoI-R primers to replace *β-actin* in the pT2-*β-actin* plasmid, and then TRE3G ligated to produce the pT2-*elf1a*-TRE3G vector.

The third step: The *NcoI* digested *rtTA*-IRES-E*gfp*-*polyA* fragment from the first step was ligated into the pT2-*β-actin*/*elf1a*-TRE3G vector from the second step. The TRE3G-*β-actin*-*rtTA*-IRES-E*gfp*-polyA fragment was then amplified with TRE3G-SwaI-F and *polyA*-HindIII-R primers and digested with *SwaI* and *HindIII*. The restriction enzyme digested fragment was cloned into a pTol2mini plasmid to generate the first generation pIDM vector.

Construction of the second generation pIDM vector:

A homologous recombination ligation method was used in the construction of the second generation pIDM vector.

The first step: the *rtTA*-IRES-E*gfp*-*polyA* fragment was amplified with a primer pair *rtTA*-IRES-E*gfp*-*polyA*-SpeI-F and *rtTA*-IRES-E*gfp*-*polyA*-EcoRV-R from the prtTA-IRES2-EGFP construct.

The second step: SwaI digested TRE3G promoter was ligated into pTol2mini plasmid to produce a pTol2mini-TRE3G vector. *β-actin* promoter was then amplified with a primer pair, *β-actin*-BglII-F and β-*actin*-NotI-R, from pT2-*β-actin*. The *elf1a* promoter was amplified from the T2KXIG plasmid with the primer pair *elf1a*-BglII-F and *elf1a*-NotI-R. The

β-actin/elf1a promoter was then ligated into a pTol2mini-TRE3G vector that was subjected to digestion with *BglII* and *NotI* to generate pTol2mini-*β-actin*/*elf1a*-TRE3G vector.

The third step: The amplified *rtTA*-IRES-E*gfp*-polyA fragment was ligated into the pTol2mini-*β-actin*/*elf1a*-TRE3G vector digested with *SpeI* and *EcoRV* to generate the pTol2mini-*β-actin*/*elf1a*-*rtTA*-IRES-EGFP-TRE3G vector. This vector had single-restriction enzyme site for *SwaI* between the TRE3G and *β-actin*/*elf1a* promoters, and for *EcoRI* between the left border of Tol2 and the SV40 terminator.

The fourth step: The core element of the chicken β-globin insulator was synthesized by the Biosune Company and cloned in a PUC57 vector. The insulator-SwaI and insulator-EcoRI fragments were respectively amplified with two pairs of primers: insulator-SwaI-F/R and insulator-EcoRI-F/R from the PUC57-insulator vector. The insulator-*SwaI* and insulator-*EcoRI* fragments were ligated into pTol2mini-β-*actin*/*elf1a*-*rtTA*-IRES-EGFP-TRE3G vectors by a homologous recombination ligation method using two separate steps.

Construction of pIDM:HA-DsRed vector:

An HA-tagged DsRed fragment was amplified from the pIRES2-DsRed vector (Clontech) with the primer pair HA-DsRed-EcoRI-F and DsRed-EcoRI-R and then ligated into a pCS2+ plasmid to obtain the pCS2+HA-DsRed vector. The HA-DsRed-polyA fragment was amplified with a primer pair HA-DsRed-polyA-AscI-F and HA-DsRed-polyA-AscI-R from a pCS2+HA-DsRed vector. The sequence of GGCGAATT downstream of the Tol2 right border in the pIDM vector was mutated to GGCGCGCC, an *AscI* restriction enzyme site, by site-directed mutation with AscI-site-mutation-F/R primers. *AscI* digested HA-DsRed-polyA fragment was then ligated into the modified pIDM vector to generate the pIDM-HA-DsRed vector.

Linker-mediated PCR for identification of pIDM insertion sites

Linker annealing was performed according to the protocol described in a previous study[2]. A cocktail containing 50 µl of 25 µM AluI or BfaI linker+, 50 µl of 25 µM AluI or BfaI linker- and 2 µl of 5 M NaCl was incubated at 95 ℃ for 5 minutes and then slowly cooled down to room temperature to allow the linkers to anneal. Annealed linkers were stored at -20 ℃. Linker-mediated PCR was performed as described previously[3]. Briefly, 3 µg genomic DNA was digested with AluI or BfaI overnight. The digested DNA was purified and dissolved in 30 µl H_2O. The Linker DNA together with the digested genomic DNA fragments was ligated with T4 DNA ligase (NEB). The ligation solution was used as the template for the first-round PCR with a pair of Linker primer and Tol2 5' N1 primer. Then the first-round PCR product was used as the template for the second-round PCR with another pair of primers: Linker AluI (BfaI) nested primer and Tol2 5' N2 primer.

After the second-round of PCR, the PCR products were purified using a PCR clean kit (Axygen) according the manufacturer's instructions and then ligated to a pGEMT-Easy vector (Promega). The Tol2 5' S1 primer was used to sequence the PCR products. The sequence of the PCR product was blasted with the database GRCz10. The sequence of genomic DNA with a high identity (>95%) to the PCR product was considered to be the insertion site. If the similarity between the PCR product and genomic sequences was less than 95%, the insertion site was considered as non-identified (N.D).

Segregation of mutation responsible for fin regeneration defect from multiple-inserted lines

From line pIDM-A28, LM-PCR amplified two insertion sites. The first insertion located in the 1st intron of *galnt2* gene with the Tet-On promoter oriented in the opposite direction (Fig. 6A). The second insertion was unable to be identified due to that the sequence of second LM-PCR amplified DNA fragment has high similarity to a number of genomic DNAs at different locations. We crossed fin regeneration defective F_1 mutants with WT to set up F_2

population. About 50% EGFP positive embryos was found in the offspring of one of these crosses, which suggested that this F_1 mutant fish may contain single insertion. Then we raised the EGFP-positive embryos from this cross to generate F_2 generation. Using a pair of *galnt2* and insertion specific primers, the insertion within the *galnt2* gene was identified in all of the F2 fish (Fig. S17A). Southern blot showed that only one insertion site was detected in the F_2 offspring from this F_1 mutant as well as the F_3 offspring from one of the F_2 mutant adult fish (Fig. S17A). The results demonstrated that all of these F_2 mutants had only one insertion within the galnt2 gene.

There were three insertion sites in both pIDM-A3 and E7. Single-inserted mutant was segregated from F2 individuals with fin regeneration defects in both lines and the EGFP positive embryos from the F2 individuals were used to generate F3 population. The single insertion was confirmed by southern blot with the F3 offspring from the F2 mutant as well as the F4 offspring from the F3 population in both lines (Fig. S17C,D). The insertion in segregated pIDM-A3 and E7 located in the 4th intron of the γ-glutamyltransferase7-like (ggt7l) gene and the 1st intron of cry61 gene with the Tet-on promoter oriented in the opposite direction (Fig. 6A), respectively. Using a pair of gene and insertion specific primers, the insertion site was identified in all of the F3 mutant fish from both of lines (Fig. S17C,D). Dox-dependent fin regeneration defective phenotype was also observed in the F3 population of both lines (54% in pIDM-A3 and 66.7% in pIDM-E7), whereas F4 EGFP positive embryos from these F3 mutants of both lines did not show Dox-dependent developmental defects (Fig. 6C), which was consistent with those in respective F1 population.

References

1 Loew, R., Heinz, N., Hampf, M., Bujard, H. & Gossen, M. Improved Tet-responsive promoters with minimized background expression. *BMC Biotechnol* **10**, 81 (2010).

2 Kotani, T., Nagayoshi, S., Urasaki, A. & Kawakami, K. Transposon-mediated gene trapping in

zebrafish. *Methods* **39**, 199-206 (2006).

3 Yergeau, D. A., Kuliyev, E. & Mead, P. E. Injection-mediated transposon transgenesis in Xenopus tropicalis and the identification of integration sites by modified extension primer tag selection (EPTS) linker-mediated PCR. *Nat Protoc* **2**, 2975-2986 (2007).

Supplementary Table S1: PCR primers

**Primers for construction of the first generation pIDM**	
rtTA-BamHI-F	CGAGCTCAAGCTTCGCCGCCACCATGTCTAGACTGGACAAGA
rtTA-BamHI-R	TACCGTCGAAAGCTTTTACCCGGGGAGCATGTCAA
rtTA-IRES-E*gfp*-*SV40*-NcoI-F	CCTCGACCATGGCCGCCACCATGTCTAGACTGGA
rtTA-IRES-E*gfp*-*SV40*-NcoI-R	CCTCGACCATGGCAGTGAAAAAAATGCT
elf1a-HindIII-F	CGAGCTAAGCTTCAGGGGGATCATCTAAT
elf1a-NcoI-R	CCTCGACCATGGCAGCTAGAACTCGCCGC
TRE3G-SwaI-F	CGAGCTCATTTAAATTTTACGAGGGTAGGAAG
polyA-HindIII-R	CGAGCTAAGCTTCAGTGAAAAAAATGCT
**Primers for construction of the secondary generation pIDM**	
rtTA-IRES-EGFP-polyA-SpeI-F	TCCTCGACGGATCCACCGGTCGCCGCCACCATGTCTAGACTGGA
rtTA-IRES-EGFP-polyA-EcoRV-R	TTGTTTAAGCTTGATGAATTCATACATTGATGAGTTTG
elf1a-BglII-F	AGCTCCTCGAAAATACAGGGGGATCATCTAAT
elf1a-NotI-R	TTCACTAGTGCGCGCGGCCGCCAGCTAGAACTCGCCGC
β-*actin*-BglII-F	AGCTCCTCGAAAATATTAGACCTTCTTACTT
β-*actin*-NotI-R	TTCACTAGTGCGCGCGGCCCTGAACTGTAAATGAATGA
insulator-SwaI-F	TGCCCAGTTTAATTTCACACTGGCGGGGAGCT
insulator-SwaI-R	GCGCGCCCTAGATTTTTCTGTCATTCTAAA
insulator-EcoRI-F	ACTCATCAATGTATGCACACTGGCGGGGAGCT
insulator-EcoRI-R	TGTTTAAGCTTGATGTTCTGTCATTCTAAA
**Primers for construction of *pIDM:HA-DsRed***	

HA-DsRed-EcoRI-F	GATCGAATTCGCCACCATGTACCCATACGATGTTCCAGATTACGCTGCCTCCTCCGAGGACG
DsRed-EcoRI-R	GATCGAATTCCTACAGGAACAGGTGGTGG
HA-DsRed-polyA-AscI-F	CGACTCACTATAGGGCGGCCGCAATTAAAAAACCTCCCAC
HA-DsRed-polyA-AscI-R	CACCTCTGGGCCCGGGCCACCATGTACCCATACGATGT
AscI site mutation-F	ACTATAGGGCGCGCCGGGCCCAGAGGTGTAAAGTACTTGAGTAATTTTAC
AscI site mutation-R	ACCTCTGGGCCCGGCGCGCCCTATAGTGAGTCGTATTACAATTCACTGGC
**Primers for the southern blot probe**	
EGFP 226 probe-F	CCCGACCACATGAAGCAGCACGAC
EGFP 646 probe-R	GCTTCTCGTTGGGGTCTTTGC
**Primers for the identification of transgenic fish**	
rtTA transgenic fish ID for	ATGTCTAGACTGGACAAGAGC
rtTA transgenic fish ID rev	TTACCCGGGGAGCATGTCAAGG
**Primers for Linker-mediated PCR**	
Linker AluI-H	CTCCCTTAAGCGGAGC
Linker AluI+H	GTAATACGACTCACTATAGGGCTCCGCTTAAGGGAG
Linker BfaI-H	TAGTCCCTTAAGCGGAG
Linker BfaI+H	GTAATACGACTCACTATAGGGCTCCGCTTAAGGGAC
Linker primer	GTAATACGACTCACTATAGGGC
Linker AluI nested primer	AGGGCTCCGCTTAAGGGAG
Linker BfaI nested primer	AGGGCTCCGCTTAAGGGAC
Tol2 5’ N1	CGCAATTCAATTGGTTTGG
Tol2 5’ N2	GCAAGGGAAAATAGAATGAAG

Tol2 5’ S1	AGTATTGATTTTTAATTGTA
**Primers for WISH**	
faf1-F	AACGCGATCTAGAACAGCAA
faf1-R	CATAGGGGCCGGTTTGAGAT
pcyt1aa-F	TGAGATCACGAATATCGAGA
pcyt1aa-R	CGTCTTCGTTCATGACAGTA
galnt2-F	GGAGGAATGCAAGATGGTTTTA
galnt2-R	AGCAAATGACTTTGGCACACTG
nidogen2-F	TTCCAAGTGGTGCTTGCGTA
nidogen2-R	CCTGGAAGTCTGGGTCGATG
grb14-F	TGCATGTGCGTGTGTGTTTTC
grb14-R	GTTTTTGGTCCAGGGTAACAG
ttyh3b-F	GGCTTCACTTGGGCTTGAG
ttyh3b-R	GAGGTGTACTCTTGCTCTGCC
sema5ba-F	CCCCTTGGATGCCAGTGAAT
sema5ba-R	TGCGTGTTCGTTGGTAGTGT
**Primers for qRT-PCR to analyze the relative levels of sense mRNA**	
β-actin-F	ACCACCGGTATTGTGCTGGATGC
β-actin-R	ACAGATCCTTACGGATGTCAATGTC
elf1a-F	CTTCTCAGGCTGACTGTGC
elf1a-R	CCGCTAGCATTACCCTCC
18sRNA-F	CGCCACTTGTCCCTCTAAGAA
18sRNA-R	GTAGTTCCGACCGTAAACGAT
faf1-F	TGACAACATTGCTGAGGCGA
faf1-R	CATAGGGGCCGGTTTGAGAT
grb14-F	TGTAAGCTGGCTCCATGAGTG
grb14-R	GTGAGCGCAATGGGATGAAG
grb14 X3-F	CGTGTACGGGAAAGAGGACG
grb14 X3-R	GGCTCGGCTGGTGTTATCTT

pcyt1aa-F	AGCAGTATCAGCGTGTCACC
pcyt1aa-R	TGTCGAAGATCCCATCAGCG
ap2b1-F	TCAACAAGCTTCTGACGGCT
ap2b –R	TTAGCATGCGAGAGACGTGG
loc556929-F	CGTGGCTCGGTCTAAACTGT
loc556929-R	ACCACGAATCCTGATTCAAGACA
plcd1a X1-F	AAAAGACAGGCAGGGCAAAC
plcd1a X1-R	ACAGCGCATGTTCAACACATC
plcd1a –F	ACGGGCTTTCGTGAAGGATT
plcd1a –R	ACCTATGCGCTTGCCATTCT
nidogen2-F	ATCAACCTGTGATAGTTCCTCCAA
nidogen2-R	GGTTGAACTCACCGGCAGA
sgcd-F	CCAGGAACAGTGCCCTCATA
sgcd-R	GTAAAGGCAACGTTTCCGCC
galnt2-F	CTTCCTCTAGCCAACCGCTG
galnt2-R	ATGCGGTGATACAGTGGCTC
si dkey-256k13.2-F	ATGCTCTGTTGTCACTCGCA
si dkey-256k13.2-R	ACATTTGAGAGCATCCCGCA
ms4a17a.8-F	CAGAGCGAAGACGCAAAGC
ms4a17a.8-R	GTGTTGGTGGTTGAGCCTGG
f2rl1.1-F	CCAGAGGTACTGGGCCATTG
f2rl1.1-R	TTGGTGACCTTCACGGTCTG
hes6-F	GAACTGCAGCAATATGGCCC
hes6-R	GCGCGTCTCTTTTTCTCCAC
mty1l X1/X2-F	CCCAGAACCCCACCGTTTTTA
mty1l X1/X2-R	CAGTCCCCGACTCTTTGGAT
myt1l X3-F	CTGTACACATGCCCGCTGA
myt1l X3-R	GCCATGACATCACGAAAGCTG
dner-F	TTAGACCGATCTGTCACCCG

dner-R	ACGTCAGAGGGGTCCCAAA
loc103909182-F	CACACTGACGGTCGTTATGC
loc103909182-R	ACGCTGGCAGTCAATCGGTA
syt10-F	GCTCGCCCTTTTGGTAGTCT
syt10-R	CATTGGACGTGAACGCCTTG
grik4-F	TGACAACATTGCTGAGGCGA
grik4-R	CATAGGGGCCGGTTTGAGAT
loc103908836-F	TTGCGGAGGTGGAATCCTTC
loc103908836 –R	GACGGCGTTTCTCTTCCTCT
grik4-002(402)-F	GATCAAAGCCACGGTTTCCC
grik4-002(402)-R	TCCCCCTCCTTTCCCTTGTT
grik4-003(532)-F	CCGCAGAAGTGTCTGTGCTA
grik4-003(532)-R	TGAACTGATAAAGGCGGCCA
loc103910266-F	TGACACGCAAGACAGTCAGT
loc103910266-R	AGTGCTTCTGCAAACCTGGA
syt17-F	CCGATCCACCTTCAGCTTGG
syt17-R	GTGTAGGTGGCTTTCGCAGA
sich211-15e22.3-F	CACATCGGAAAACATCGCCC
sich211-15e22.3-R	GCGACTTCTCGTTACCCCAA
acbd5a-F	ACTTCTGGGCTTGGCTTGAC
acbd5a-R	ACCGCTTCAAGAAGATCCGAA
abi1a-F	GGTTCAAAAGAACGACTCCGA
abi1a-R	AGCTTCCTACTCCTCGTCTGT
TANC1-like-F	GGAGAAGGGTGCTGTGATCG
TANC1-like-R	AGGAGACGTTCTGTAGCCTAGT
Loc103911696-F	CACAGGGCTACTCCAGAACG
Loc103911696-R	CTTCCTCAAGACTCGACCGC
evi5a-F	TTGGAGAGGAGGTGCTCTGA
evi5a-R	AGCTGATTAGCTCTGTCCGC

Loc101886424-F	TGTCTTGTTATTAAAAGCTATGGGC
Loc101886424-R	GCCGGAAGATTGGATGTGG
wu:fc21g02-F	TTGGTGGGCATCAGAAAGACA
wu:fc21g02-R	GCCATGGTCTATAGCTTTGCG
tcrig1-F	TGTCGTGGCAAGGCTATGTT
tcrig1-R	TCCCTCCATAACCAGCAGGA
Loc101882854-001-F	ACACCGTATGGTTGATAACTGC
Loc101882854-001-R	ATCCAAGCAACCCACCACTT
Loc101882854-002-F	ACACCGTATGGTTGATAACTGC
Loc101882854-002-R	ATCCAAGCAACCCACCACTT
scn8aa-F	AACCGGTGCTACCTATTGGC
scn8aa-R	TGCGGATGTCGCCATATTGT
*tmprss*12-F	CATCACTGGATGGGGCAGTT
*tmprss*12-R	ACATGGCCATTATGCCACCA
NLRC3-like-F	GCTGTGATCTGACCAAGCCT
NLRC3-like-R	GAAGCCACGAACCTCCATGA
snx17-F	TTTTTCCGTAGCCGTTCGGT
snx17-R	AGGAGTGAAGACAGTTTGCCC
prpf18-F	TGTGGATACAAGATGGACAAACCT
prpf18-R	CGTCTGATCACCTCCTGTCG
ogdhb-F	GCATCGCTTAAGGACTTGCG
ogdhb-R	AGTTGGAGCTCGTCCCATTG
gpr128-F	CTGCCAGCCGTTATTGTTGG
gpr128-R	GCCAGCCAGCAAAACTCTTC
picalmb-F	CACGAGCTTTGTCTCGGTCT
picalmb-R	TAATGCTCTGCCCGGACATC
shisa-7-like-F	TGCCACAACTACCAATCCCC
shisa-7-like-R	TCCACCAATGACGTACACGG
efnb1 A-F	CAAGAGAACCCGGAAGCACT

efnb1 A-R	ATTCTCTGTTGTCCGCAGGG
efnb1 B-F	GAAGTGACGGCTCGTGGAA
efnb1 B-R	ACCGACTCCAGTGATTTGGC
loxl2a-F	AACGCAAGCATTACGAGGGT
loxl2a-R	AGTGGACATTGTCGAGCCAG
tbx1-F	CAAGCTCAACGCCGAACAAA
tbx1-R	TCTGCCATTGGGTCCATTCC
ablim3-F	CAGGCTTGCTTTACCCGAGA
ablim3-R	TGATAATGGGAACGGTGGGC
dapk1 A-F	ACAAGCATGGAACTCCTCCG
dapk1 A-R	CTCAAGGTCCCCACATGACC
dapk1 B-F	ATCACCCTGCACGATGTGTT
dapk1 B-R	GGCTTCAGGTCAAAGTGGGA
ebag9-F	CAGATCACCCTCCCAACCAC
ebag9-R	CCTGATGGTTGGAGCCATGT
matn4-F	GGACTGTTCACTCCATGCCT
matn4-R	GACGCAGGAATGCTTCACAG
prdm5-F	TCCACAGTGAGGACAAACCG
prdm5-R	CGCTGTAAAGAGAACGGGGT
ube2nb-F	TCAACCTCAACGCGAGGAAC
ube2nb-R	ATAATCCTGCGCGGCAATCC
tbc1d8-F	AGGTTTGATTGCGGAGGAGG
tbc1d8-R	AGGAGTAATGCACCACCAGC
ifnphi3-F	GTGCAGGTTTGGTCATTGCC
ifnphi3-R	ATAGAAACGCGGTCGTCCAG
sema5ba-F	CACTCAGGAATGTGCCAAAGG
sema5ba-R	AGCAGAGTGAACCCTGGACA
plekha5-F	GTCCAGGCCTCAGTTCAACA
plekha5-R	AGCAAACGATGAAGGCCAAC

solute carrier family 45 member 4-like-F	ATGATCCACGAAGACGAGCC
solute carrier family 45 member 4-like-R	TGGCTAAAACGGAGTCGCTT
lhfpl4-F	TGTTCTGTTCGCTGGGCTT
lhfpl4-R	CTCGGATCTCAATCCTGGATAC
nsmce1-F	TCAACACTCACCTTCAGCCC
nsmce1-R	CACAATCAGATCCATCAAAACAAAA
sfswap-F	TATACTTCGCCTTTCCCGCC
sfswap-R	TCCACAGGACACGCCAAAAT
itcha-F	CTGCTAAGCTGCGGGAGAAT
itcha-R	CTTCGACTGTCCGTCCACAA
anti-faf1 qRT-For	tttagcctccaccagattcc
anti-faf1 qRT-Rev	gacgagtttaaactgctcac
anti-grb14 qRT-For	ctgaaacttcctcctatgc
anti-grb14 qRT-Rev	gaaccgttgatacaaggca
**Gene specific primer for *in vitro* reversely transcribing sense-cDNA**	
β-actin sp primer	TGTACGGCTGAGAGACTGAG
faf1 sp primer	TTAGGGGAGGTTTAGGGCAGT
nidogen2a sp primer	AAACCAGACGCTTCCCATCC
grb14 sp primer	TTAGTTCGCGCAGACAGAAGT
sgcd sp primer	GGGGCCCACGAGGATAAAAA
ap2b1 sp primer	GCATTGTAGTCCCCGAGACA
**Gene specific primers for qRT-PCR to analyze antisense transcripts**	
faf1-intron6-F	ACAAATGGCACAAACCCTTTACA
faf1-intron6-R	TGCTCGGTCGCTTACTTTGT
grb14-intron2-F	AGGACATCAGGGAGGTCAGG
grb14-intron2-R	TAAAAGCGTCTTGCCCCGAA
galnt2-intron1-F	GTAGGTGGCACTGGGATGTT

galnt2-intron1-R	TACACCCCTCTTCCAGCAGT
nidogen2-intron9-F	TGACTGCCCTTGCCTATTCC
nidogen2-intron9-R	GTTGGGCTGAGTAGCTGGTT
sgcd-intron2-F	GTGTCCAGAGGGTTTACGGG
sgcd-intron2-R	CTGTAGCTGCCGCACAATTC
ap2b1-intron21-F	GTATTTCCAGGGGTCTCGGC
ap2b1-intron21-R	CGCGATCAGAAACCCTACCT
**Primers for analyzing morpholino function**	
nidogen2a-MO detection for	CAGCTCTAGCCCATCCCATC
nidogen2a-MO detection rev	GGTTCTTGACGGACTGCTCA
grb14-MO detection for	TCACACTGCTGCCTGTACTT
grb14-MO detection rev	TGGATATCGACACCATGTGCT
pcyt1aa-MO detection for	AAATCGCCGTACACTTCCGA
pcyt1aa-MO detection rev	GTCGAAGATCCCATCAGCGT
**Primers for analyzing insertion sites**	
galnt2 ID for	GCAAGGGAAAATAGAATGAAG
galnt2 ID rev	GTTCGCTTTGGGTGGGCA
ggt7l ID for	GCGATGCCACCTACGGCA
ggt7l ID rev	TGCTTAAACCATGAGGACAGGG
cyr61 ID for	GGACGACGGCAACTACAAGA
cyr61 ID rev	ATACCTCAAAGCAACTGTCCCA
*ttyh3b*ID for	GCAAGGGAAAATAGAATGAAG
ttyh3b ID rev	GGTTGTGAAAGCGTGCCTA
si:dkey-26i13.8 ID for	GCAAGGGAAAATAGAATGAAG
si:dkey-26i13.8 ID rev	GCTCTGAGGGGATGGCTTG
**Primers for identification of *nid2a* cas9-mutant**	
nid2a cas9 ID for	GCAGGTGACCACCAATGGAA
nid2a cas9 ID rev	CCTCAAAGCTGTTTTCTTACCCG
**Primers for constructing ubi-pIDM vector**	

TRE3G-F	CTGATGCCCAGTTTAATTTGGCG
TRE3G-F	gatgatcaatCTGCAGAAAATTCGAGGAGCTT
4XIns-F	aagctcctcgaattttctgcagATTGATCATCTGCAGATCATCACACTG
4XIns-R	tggtCTTGTTTATTGGGCCCACTAGAT
Ubi-F	tagtgggcccaataaacaagACCAGCAAAGTTCTAGAATT
Ubi-R	ttaaatggaaatgtgtgattgctaatcttactttgaatttgtttacaga
rtTA-F	atcttactttgaatttgtttacagaccggtGCCACCATGTCTAGACTGGAC
rtTA-R	ACCGCATGTTAGCAGACTTCCTCTGCCCTCCCCGGGGAGCATGTCAAGGT
T2A-RFP-F1	TAACATGCGGTGACGTCGAGGAGAATCCTGGCCCAATGGCCTCCTCCGAGGACGT
T2A-RFP-F2	gagggcagaggaagtctgctaacatgcggtgacgtcgaggagaatcctggccca
T2A-RFP-R	cttatcatgtctggatcatcatcggctagcCTACAGGAACAGGTGGTGGCG
**Primers for Tail-PCR**	
R1	GGCCCAATAAACAAGAATCTCTAGTTTTCTTTCTTGCTTTTAC
R2	ACGATGGACTCCAGTCCGGCCTAATACTCAAGTACAATTTTAATGGAGTAC
R3	TTTCCCTAAGTACTTGTACTTTCACTTGAGTAA
L1	CGCGCCAAATTAAACTGGGCATCAGCGCAA
L2	acgatggactccagTCCGGCCTAGAATGAAGTGATCTCCAAAAAATAAGTA

L3	ACTTAAGTACAGTAATCAAGTAAAATTACTCA AGTAC
LAD1-1	acgatggactccagagcggccgcvnvnnnggaa
LAD1-2	acgatggactccagagcggccgcbnbnnnggtt
LAD1-3	acgatggactccagagcggccgcvvnvnnnccaa
LAD1-4	acgatggactccagagcggccgcbdnbnnncggt
AC1	acgatggactccagag
**Primers for cloning transgenic insertions**	
LP3	AAATAGAATGAAGTGATCTCC
RP3	CTCAAGTAAGATTCTAGCCAG
6-GR-1	AAGTTGCGTGAAATGCACAA
6-GL-1	ATGCTGGTTAGTTTACAGA
6-GL2	AAACCTTGAGGTGTGCGTTA
6-GR2	ATACACTACCACAACTACTA
6-GL3	TGCCACAGAAGAATGAACCG
6-GR3	TATTTAGGTGGTCCGGTGG
8-GL1	GTCAGCAAAGAACCTGCGAC
8-GR1	ATGAGGTCATTCGTCTGCGAT
8-GL2	GCATTAAGCTGTGTCGTCAGC
8-GR2	GTAGACATGACAAGTTAAAT
8-GL3	ACACCAAAACCCAACACACA
8-GR3	ATAAATTGCTTGTTTTAAAACG
13-GL1	CATGGTCTGCTGAGAGCGAG
13-GR1	AGGAGAGCGTCTGCAACATC
13-GR2	GCGTATTCGCAAAGTGACAT
13-GL2	ACAAAAAAATGTTAGACGTTCTGGA
23GR1	TTTTCCTTCAATGACCTATT

23GL1	TGGGCCAGATTACCAACCAG
23-GR2	TTCGATGACTGATACAGATTGTGT
23-GL2	ACCCAGTTAGGATAGCTGATT
37-GL1	GTCCAAAACGAGTCAGCGATGG
37-GR1	ACGTTAGTAGTAAGGTGCTA
37-GR2	GAGGGAGGTAGAGCTGCCCT
37-GL2	ACTGTGACAAAGGCTCAGCT
37-GL3	GAAACATGCCAAGTGAACAG
37-GR3	TACATGACATTTATTGCCAT
37-GL4	ATCCCCATAGACAAGAAAAA
37-GR4	CTCTCTCAACCTGTTAAATT
43-GL1	CTGAATGCCCTGCAGCCCTGACA
43-GR1	GTGTGGACGGAGATATGTTC
43-GL2	CTGAATGCCCTGCAGCCCTGACA
43-GR2	GTGTGGACGGAGATATGTTC
43-GL3	ATTTTTGACAAGCACCATAAA
43-GR3	CTTGATCTGTTGTTTATGCA
84GR1	TATTTGACCATGTGTGTTATA
84GL1	GGCTGGTGGCTGAGTGAAAT
84-GL2	CAATGCATAGGCAGACAGTGTC
84-GR2	AAAGGTCATAGTTTGGTTTCAGTT
84-GL3	CCGTCTCCGCTCTCCAGGTGCAGTT
84-GR3	TAAACCTAACCGTCATTGGGTGATG
84-GL4	AGCAACTCTGCGTAAACAAACGCT
84-GR4	CTAGTAGACTTACGTGAGAA
**Primers for generating RNA probes**	
dnajbF1057Spe	TGCGGCAAACCGGCATCATT
dnajbR1057spe	GGAAAGGCAGGCCTTCTCCA

proza-F	TCCTCCGTCATCATGGCTTG
proza-R	ACACTCTCTAGAGTTTCCCATCAA
pE3-ST6-F1130	GACTTAATTACACAGCCATT
pe3-ST6-R1130	GTGAAATAACTGACTTAGCTGT
SPS2 F-1111spe	CAAATGTCCTGAGTGATCTG
SPS2 R-1111spe	ATTTTTGGTCTGTTGCTCTC
